# CDAF: a co-evolutionary decoupled attention framework for explainable weak thermal fault diagnosis of marine diesel engines

**DOI:** 10.1038/s41598-026-46425-w

**Published:** 2026-04-14

**Authors:** Zaimi Xie, Chunmei Mo, Baozhu Jia

**Affiliations:** 1 Naval Architecture and Shipping College, Guangdong Ocean University, Zhanjiang, 524088 China; 2 Technical Research Center for Ship Intelligence and Safety Engineering of Guangdong Province, Zhanjiang, 524088 China; 3 Guangdong Provincial Key Laboratory of Intelligent Equipment for South China Sea Marine Ranching, Zhanjiang, 524088 China; 4 College of Electronic and Information Engineering, Guangdong Ocean University, Zhanjiang, 524088 China

**Keywords:** Marine diesel engine, Weak thermal fault diagnosis, CRIME algorithm, Dual-path feature decoupling, Adaptive attention mechanism, Explainability, Engineering, Mathematics and computing

## Abstract

As marine diesel engines serve as the primary power source for ships. Traditional intelligent fault diagnosis methods fundamentally struggle to extract weak drift trends masked by strong noise, lacking adaptive parameter optimisation prevents the accurate delineation of fuzzy health-state boundaries in high-dimensional space, as well as poor explainability due to the black-box dynamic fusion of multi-source parameters during inference. This paper develops a novel Co-evolutionary Decoupled Attention Framework (CDAF). Driven by the Cooperative Rime Information Migration Evolutionary (CRIME) optimisation algorithm, to overcome the local minima inherent in manual tuning, the framework seamlessly integrates physically-informed dual-path feature decoupling (DPFD) to isolate weak drifts in high-noise environments, and employs a joint channel-spatial attention mechanism to resolve the fuzzy boundaries of highly similar fault states in high-dimensional space and accurate multi-source feature fusion. Fusion-CAM is further introduced to establish ensure interpretability throughout the inference process, which quantifies branch contributions of the fusion layer and maps thermal parameter responses to engine health status. Validation using real-ship and simulation data shows that the proposed method achieves 99.71% and 95% accuracy under standard and high-noise conditions, respectively. DPFD and CRIME modules enhance accuracy by over 2.2%, demonstrating the value of feature decoupling and global optimisation. Furthermore, Fusion Class Activation Mapping (Fusion-CAM) outperforms Grad-CAM + + in visualization accuracy, as confirmed by feature occlusion tests. Fusion-CAM quantitative analysis reveals that the weight of the trend-guided path exceeds that of the average path. This indicates that parameters such as maximum pressure during combustion, maximum temperature during combustion, brake mean effective pressure, exhaust gas pressure after the turbocharger, and exhaust manifold temperature are critical indicators for diagnosing subtle intake valve leakage.

## Introduction

 As the primary power source for marine propulsion, the safety and maintainability of marine diesel engines are fundamental to the operational reliability of vessels^1^. The intricate internal architecture and tight component coupling require robust Prognostics and Health Management (PHM) strategies to prevent catastrophic failures and ensure mission integrity^2^. As a pivotal component of PHM, thermal fault diagnosis provides actionable insights for maintenance decision-making, effectively preventing unplanned shutdowns and enhancing the safety and reliability of diesel engine structures, systems, and components. More broadly in complex industrial systems, precise fault classification and cause reasoning serve as the fundamental basis for advanced decision-making, such as implementing robust fault-tolerant control^3^. This is especially critical during deep-sea navigation, marine diesel engines inevitably operate in dynamic, non-stationary states due to the continuous superimposition of environmental factors, including high-salinity fog, severe pitching, marine corrosion, and sudden load fluctuations^4^. Such unstable operating conditions disrupt key thermal processes, including in-cylinder combustion and heat transfer, triggering a chain reaction that ranges from fluctuations in intake efficiency to imbalances in fuel atomisation, ultimately destroying combustion consistency. These deviations across the full thermal cycle may induce, weak thermal faults, which manifest as slow, latent drift in thermal parameters. However, traditional vibration-based fault diagnosis methods primarily target mechanical faults and rely on post-event analysis. Consequently, they possess limited predictive capabilities and lack the sensitivity required to detect subtle anomalies or elucidate the evolutionary trajectory of incipient, slow-varying thermal faults^5^. Therefore, developing explainable data-driven models based on thermal parameter monitoring has emerged as a vital necessity. Such approaches must not only capture the subtle trends of weak faults but also provide a physically traceable link between feature representations and the engine’s health status, satisfying the strict demands for intelligent monitoring of marine diesel engines.

To address these challenges, research has evolved from fundamental signal analysis to sophisticated deep mixed representations. Early studies utilised Kalman filters^6^, recurrence plots^7^, and entropy theory^8^. However, these methods struggle to isolate weak features submerged in heavy noise. The advent of artificial intelligence has introduced CPLA-ICEEMDAN^9^, Convolutional Neural Networks (CNNs)^10^, Long Short-Term Memory Networks (LSTMs)^11,12^, and wide-convolution variants^13,14^ for anti-noise diagnosis. Concurrently, advanced data-driven models like deep residual frameworks have proven highly effective for quality prediction and nonlinear information extraction in complex industrial processes^15^. While such frameworks provide a robust foundation, the unique operating environments of marine diesel engines pose distinct challenges, necessitating models that can characterize highly coupled thermodynamic nonlinearities. Consequently, researchers have developed hybrid architectures, such as the Kolmogorov–Arnold Network integrated CNN and Gated Recurrent Unit (CNN-GRU-KAN)^16^, Thermodynamic Simulation-aided Random Forest (TSRF)^17^, and the Dense Connectivity Neural Network (DCNN)^18^. Despite these advancements, existing hybrid models remain inadequate for accurate decoupling and fine-grained representation of deep features, given the unique slow-variation characteristics of marine thermal parameters. Because weak thermal faults lack high-frequency mutational energy and manifest as hysteretic drifts, they are easily masked by stochastic noise.

Furthermore, high-dimensional nonlinear coupling between the combustion, intake, exhaust, and cooling subsystems induces feature aliasing, preventing models from precisely extracting deep, slowly varying features. While channel^19,20^ and spatial attention mechanisms^21–24^ have been introduced to alleviate this, they often neglect internal coupling between dimensions and rely on additional parameter layers that increase computational overhead. Moreover, global-pooling-based compression in these methods frequently results in the loss of subtle fault symptoms, making it difficult for the model to effectively attend to weak fault-related features.

As network architectures grow in complexity, generalisation increasingly depends on a vast hyperparameter space, rendering manual tuning inefficient. Although Bayesian optimisation^25^, particle swarm optimisation^26^, and the Rime Optimisation Algorithm (RIME)^27^ have been proposed, metaheuristic approaches such as RIME and its variant SRIME still suffer from slow convergence and a tendency to become trapped in local optima^28^. Furthermore, to bridge the black-box gap in deep learning, visualisation techniques such as Grad-CAM^29^, Grad-CAM++^30^, and Score-CAM^31^ have been utilised to interpret internal decision logic^32^. However, these are primarily designed for single-link structures and lack the capacity to analyse multi-branch generalised multi-source feature fusion mechanisms. Overall, existing methods fundamentally struggle to extract weak drift trends masked by strong noise, lacking adaptive parameter optimisation prevents the accurate delineation of fuzzy health-state boundaries in high-dimensional space, and the opaque dynamic fusion of multi-source parameters. It makes it exceedingly difficult to establish a highly accurate, rapidly responsive, and reliable diagnostic framework.

Aiming at the above problems, this paper develops an explainable weak thermal fault diagnosis method for marine diesel engines, named Co-evolutionary Decoupled Attention Framework (CDAF). Firstly, a novel co-evolutionary Decoupled Attention Framework (CDAF) is proposed for marine diesel engine fault diagnosis. Driven globally by the CRIME algorithm for dynamic hyperparameter optimization, to overcome the local minima inherent in manual tuning, the framework initially utilizes a physically-informed Dual-Path Feature Decoupling (DPFD) module is initially utilized to isolate weak drifts from noisy environments, and subsequently employs a Joint Channel-Spatial Attention Mechanism (JCSAM) to resolve the fuzzy boundaries of highly similar fault states in high-dimensional space and accurate multi-source feature fusion. Finally, Fusion-CAM is deployed to guarantee full inference interpretability by quantifying fusion branch contributions and mapping thermal responses to corresponding health states. The main contributions of this paper are summarized as follows:

1. A novel Co-evolutionary Decoupled Attention Framework (CDAF), dynamically optimized by the CRIME algorithm, is proposed for weak thermal fault diagnosis. By embedding a physically-informed Dual-Path Feature Decoupling (DPFD) module, the framework effectively isolates incipient thermodynamic degradation drifts from high-intensity, stochastic sea-state noise.
2. A Joint Channel-Spatial Attention Mechanism (JCSAM) is designed for transparent feature fusion. By adaptively recalibrating weights across both channel and spatial dimensions, this mechanism successfully delineates the fuzzy boundaries of highly intertwined health states in the high-dimensional feature space.
3. A comprehensive interpretability evaluation scheme based on Fusion-CAM is established. It fundamentally breaks the “black box” nature of dynamic multi-source parameter fusion by quantitatively mapping local thermal parameter responses to the global engine health status, ensuring highly reliable diagnostics.

The remainder of this paper is organised as follows. “Proposed methods” details the architectural components of the co-evolutionary decoupled attention framework, covering methodologies for physically-informed dual-path feature decoupling, joint channel-spatial attention mechanism, co-evolutionary optimization based on CRIME, and Fusion-CAM visualisation. “Experimental study” presents a comprehensive evaluation and discussion of the framework’s performance, using a custom-built dataset of weak thermal faults for rigorous validation. Finally, “Conclusion” summarises the core findings and offers concluding remarks.

## Proposed methods

### Co-evolutionary decoupled attention framework (CDAF)

#### Physically-informed dual-path feature decoupling (DPFD)

**Fig. 1:**
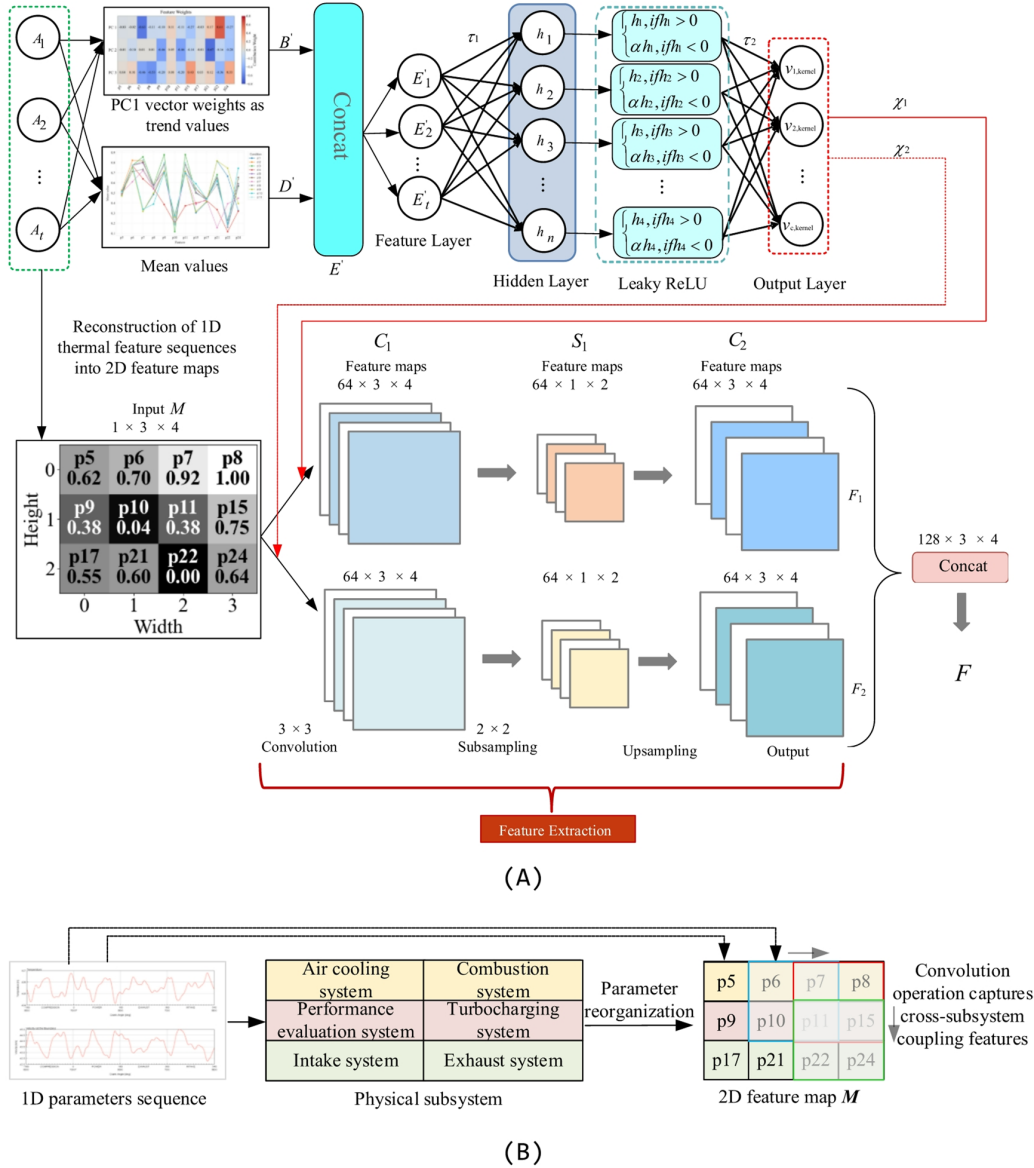
Overall architecture of the DPFD and its physically informed parameter mapping. (**A**) DPFD module, (**B**) mapping 1D thermal parameters to a 2D feature map via row-wise subsystem aggregation.

The selection of thermal parameters follows the screening framework established in our prior work^33^. Unlike previous studies, the current investigation focuses on incipient and slowly varying fault characteristics under high-noise operating conditions, specifically exploring the interaction between the screened parameters and the proposed dynamic filtering network. The original data matrix *X*$\in {\mathbb{R}^{1 \times m}}$ is processed via this framework to yield the refined matrix${X}^\prime$. Subsequently, feature extraction is performed on these selected parameters to uncover latent spatial feature representations. As a cornerstone of feature extraction, the Convolutional Neural Network has demonstrated superior representational capabilities in complex data modelling^34^. Despite their strengths, standard CNN modules often struggle to extract subtle drift features typical of diesel engine thermal parameters. To address this, we developed a physically-informed Dual-path Feature Decoupling (DPFD) module, integrated within the standard CNN architecture, to suppress high-frequency noise and accurately capture subtle drift features. In this module, the temporal trend and local mean of the features are utilised to guide a Multi-Layer Perceptron (MLP) in dynamically generating convolutional filters; the detailed architecture is illustrated in Fig. 1A. Subsequently, a sliding window technique is employed to augment the training samples^35^. The preprocessed data matrix ${X}^\prime$ is partitioned into successive segments,$A \in {R^{w \times m}}$, using a window of size *w* and a unit step size (stride = 1), where each segment *A* serves as an individual input sample. To further empower the network to capture latent, slowly evolving fault dynamics, Principal Component Analysis (PCA)^36^ is applied to each matrix segment *A*. By extracting the first principal component, we derive the trend feature matrix $B \in {R^{1 \times m}}$, as formulated in Eq. (1):

1 $$Cov({A_t})B = \lambda B$$

Specifically, the covariance matrix $Cov({A_t}) = \frac{1}{w}{A_t}^T{A_t}$ is computed from the input segment, and the eigenvector ${B_1} \in {\mathbb{R}^m}$ corresponding to the largest eigenvalue ${\lambda _1}$ is extracted as the first principal component. This vector identifies the direction of maximum variance within the thermal parameter data, effectively capturing the dominant evolutionary trend of the windowed sequence ${A_t}$.

Furthermore, the local mean of the matrix segment ${A_t}$ is computed by averaging the feature maps across the *w* time steps within the window. This operation yields a steady-state feature representation, $D \in {\mathbb{R}^{1 \times m}}$, that captures the aggregate statistical characteristics of the current window, as formulated in Eq. (2).

2 $$D = \frac{1}{w}\sum\limits_t^w {{A_t}}$$

In this formula, ${A_t}$ denotes the thermal parameter data associated with the *t*-th window segment, while *D* represents the arithmetic mean of the corresponding matrix segment.

Furthermore, the trend feature matrix *B*_1_ and the local mean matrix *D* are restructured into two-dimensional matrices, ${B^\prime} \in {\mathbb{R}^{1 \times {m_1} \times {m_2}}}$ and each with two dimensions ${m_1} \times {m_2}$, based on the established thermodynamic logic of the diesel engine. In this mapping, the magnitude of each element corresponds to a specific pixel intensity, thereby transforming the raw vector data into a series of trend-based and mean-based grayscale feature maps. These dual-domain feature maps, representing both dynamic evolution and steady-state characteristics, are concatenated along the channel dimension to construct the feature tensor *E* of size 2 × *m*_1_ × *m*_2_. This tensor serves as the high-dimensional input for the subsequent improved dynamic filtering module, as defined in Eq. (3).

3 $$E = \mathrm{Concat}({B^\prime},{D^\prime})$$

The feature tensor *E* integrates the static magnitudes and dynamic patterns from the original window; it is then projected into an ${m^\prime} = 2 \times {m_1} \times {m_2}$ dimensional feature vector ${E^\prime}$ via a flatten operation, providing a unified representation for the filter generation network. To balance computational efficiency with nonlinear modelling capacity, a MLP^37^ is employed to synthesise the dynamic filters. This network ingests the fused feature vector ${m^\prime}$ and, through the nonlinear mapping of its hidden layers, generates the *o*th high-dimensional kernel vector, ${\Upsilon _o}$, as expressed in Eq. (4). The integration of the Leaky ReLU activation function mitigates the neuron death phenomenon associated with standard ReLU functions, thereby ensuring the numerical stability and robustness of the generated filters.

4 $$\begin{array}{l}{\Upsilon _o} = {\tau _2} \cdot \mathrm{LeakyReLU}({\tau _1} \cdot {E^\prime} + {b_1}) + {b_2},\\\mathrm{LeakyReLU}({E^\prime}) = \left\{ \begin{array}{l}{E^\prime},if{E^\prime} > 0\\\alpha {E^\prime},if{E^\prime} < 0\end{array} \right.\end{array}$$

In this formula, ${\tau _1}$, ${\tau _2}$, ${b_1}$, and ${b_2}$ denote the weights and biases of the first and second layers, respectively, which are all learnable parameters of the MLP. Furthermore, α is a small constant, typically set to 0.01 or 0.1, representing the negative slope coefficient of the Leaky ReLU activation function, which ensures gradient flow for negative input values.

Subsequently, to ensure the output vector *v*_kernel_ aligns precisely with the parameter requirements of the target convolutional operator, the model dynamically reshapes *v*_kernel_ to the required kernel dimensions. Specifically, it is transformed into a dynamic filter $K \in {\mathbb{R}^{{C_\mathrm{in}} \times {C_{\mathrm{out}}} \times k \times k}}$, where *C*_out_ and *C*_in_ represent the output and input channel dimensions, respectively, and *k* denotes the spatial kernel size. This window-specific convolution kernel is then applied to the input feature map *M* via a standard convolutional operation. To intuitively demonstrate the mapping from physical information to data structure, a schematic diagram of the parameter reorganization is presented in Fig. 1B. Crucially, the reconstruction of the 12 one-dimensional thermal parameter sequences into a 3 × 4 2D spatial matrix, defined as the initial feature map *M*, is not arbitrary; it strictly adheres to the thermodynamic topology and energy flow of the marine diesel engine. Column-wise, the left field aggregates the intake system and performance evaluation system, while the right field aligns the high-temperature gas dynamics. Row-wise, by spatially grouping the air cooling system (p5, p6) and combustion system (p7, p8) parameters in the top row, aligning the performance evaluation system (p9–p11) and turbocharging system (p15) parameters in the middle row, and placing the intake system (p17) and exhaust system (p21, p22, p24) dynamics in the bottom row, the sliding *k* × *k* convolutional kernel traverses the feature map ***M*** in both directions: sliding horizontally to extract localized, intra-subsystem parameter variations, and sliding vertically across rows to naturally capture the cross-subsystem thermodynamic coupling of the engine’s air-gas loop. This physically-informed construction of feature map *M* enables the dual-path CNN to efficiently decouple weak fault features from random sea-state noise, ensuring that the extracted representations possess strong interpretability and diagnostic reliability. This adaptive mechanism enables the effective extraction of latent fault features distributed across varying operational states, resulting in the refined feature map *F*, as defined in Eq. (5).

5 $$\left\{ \begin{array}{l}{F_{o,ij}} = \Phi ({(M \cdot {\Upsilon _o})_{ij}}) = \Phi (\sum\limits_c {\sum\limits_{{v_3}} {\sum\limits_{{v_4}} {{M_{c,i + {v_3},j + {v_4}}} \cdot {\Upsilon _o}({v_3},{v_4}) + {b^\prime})} } } \\F = \mathrm{concat}({F_1},{F_2})\end{array} \right.$$

In this expression, *M* represents the input feature map, while ${\Upsilon _o}$ denotes the dynamic convolution kernel explicitly synthesised for the current window. The variables *v*_3_ and *v*_4_ signify the row and column spatial offsets of the kernel, respectively, and *c* denotes the feature map channel. Furthermore, ${b^\prime}$ represents the learnable bias and ${F_{o,ij}}$ refers to the pre-activation response at position (*i*, *j*) of the output feature map. The nonlinear activation function, $\Phi$, such as the Rectified Linear Unit (ReLU), subsequently processes this response, to generate the final activated feature map. Following the independent extraction processes, the feature maps from both pathways, denoted as ${F_1},{F_2} \in {\mathbb{R}^{64 \times 3 \times 4}}$, are fused along the channel dimension. The concatenation operation that yields the comprehensive fused feature tensor *F*. This concatenated tensor organically integrates the macroscopic degradation trends with the microscopic operational fluctuations, establishing a robust foundation for the subsequent co-evolutionary adaptive attention mechanism.

#### DPFD-JCSAM

**Fig. 2:**
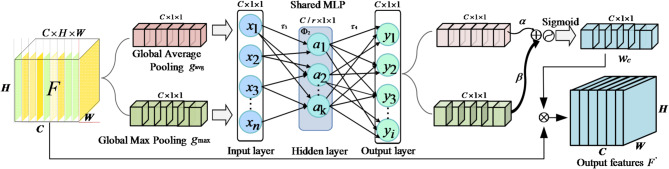
ACAM module.

Following the acquisition of the feature map $F \in {\mathbb{R}^{C \times H \times W}}$ via the DPFD module, it is recognised that not all *C* feature channels contribute equally to the diagnostic task. To further address the challenge that the fused features *F* of normal and early weak fault states are highly similar and intertwined in the high-dimensional space (i.e., exhibiting fuzzy boundaries), a Joint Channel-Spatial Attention Mechanism (JCSAM) is implemented after the convolutional fusion layer. The JCSAM comprises an Adaptive Channel Attention Mechanism (ACAM), dedicated to adaptively recalibrating key feature dimensions, cascaded with an Improved SimAM (ISimAM) network designed to precisely pinpoint local anomalies within the 2D physical topology. Unlike traditional channel attention mechanism^38^, which often suffers from weight bias under intense noise, the ACAM adaptively recalibrates channel-wise responses to mitigate this issue. By dynamically amplifying salient thermal deviations while suppressing redundant or noise-perturbed components, it ensures the precise extraction of subtle fault signatures from complex non-linear couplings. The ACAM architecture comprises three primary stages: Squeeze (information compression), Excitation (weight generation), and Recalibration (feature adjustment); the detailed structure is illustrated in Fig. 2. The process begins with the Squeeze operation, which aggregates global spatial information. To capture a more comprehensive statistical representation of each channel, Global Average Pooling (GAP) and Global Max Pooling (GMP) are executed in parallel. These operations compress each 2D feature map of size $H \times W$ into distinct scalar descriptors. For the input feature map *F*, this dual-pooling strategy yields two *C*-dimensional channel description vectors, ${g_{avg}}$ and ${g_{\max }}$, representing the average and peak spatial responses, respectively, as formulated in Eq. (6).

6 $$\left\{ \begin{array}{l}{g_{avg}} = \frac{1}{{H \times W}}\sum\limits_{i = 1}^H {\sum\limits_{j = 1}^W {{F_{c,i,j}}} } \\{g_{\max }} = \mathop {\max }\limits_{1 \le i \le H,1 \le j \le H} {F_{c,i,j}}\end{array} \right.$$

Subsequently, the module enters the weight excitation phase. The two-channel descriptors, ${g_{avg}}$ and ${g_{\max }}$, are fed into a shared MLP with a bottleneck architecture, designed to capture complex, nonlinear inter-channel dependencies while minimising parameter overhead. Following an element-wise summation ($\oplus$) of the dual-path outputs, the aggregated signal is processed by a Sigmoid activation function (${\Phi _1}$). This transformation maps the values into a normalised range, yielding the final channel attention weight vector. ${w_c} \in {\mathbb{R}^C}$, which represents the relative importance of each feature map. This process is formally defined in Eq. (7).

7 $${w_c} = {\Phi _1}(\alpha \cdot {\tau _4}{\Phi _2}({\tau _3} \cdot {g_{\mathrm{avg}}}) + \beta \cdot {\tau _4}{\Phi _2}({\tau _3} \cdot {g_{\max }}))$$

Specifically, α and β are two learnable weighting coefficients that respectively modulate the contributions of the global thermodynamic background (mean value) and the instantaneous fault-induced response (maximum value). When a weak fault is detected, the system adaptively increases the gain of the β pathway, allowing the abnormal peak responses captured by max pooling to dominate the final channel-wise weights. ${\Phi _2}$ denotes the internal activation function, implemented as a Rectified Linear Unit (ReLU), facilitating the connection between the fully connected layers. The terms ${\tau _3}$ and ${\tau _4}$ represent the weights of the two fully connected layers, respectively. To improve computational efficiency, a dimensionality reduction ratio, $\gamma$, is introduced, resulting in weight matrices with dimensions defined by $\mathbb{R}^{{C}/{\gamma} \times C}$. Finally, through the feature weight recalibration mechanism, the learned channel attention vector ${w_c}$ is applied to the original feature map *F* via channel-wise multiplication. This process yields the final output feature map, ${F^\prime}$, which contains enriched, high-discriminative information, as expressed in Eq. (8).

8 $${F^\prime} = {w_c} \otimes F$$

In this operation, $\otimes$ represents element-wise multiplication facilitated by channel-wise broadcasting. Guided by the weight vector *w*_*c*_, the mechanism selectively amplifies channels containing high-discriminative information while attenuating those with redundant or noise-related features. This adaptive recalibration ensures that the most relevant thermal characteristics are prioritised for the subsequent diagnostic stages.

**Fig. 3:**
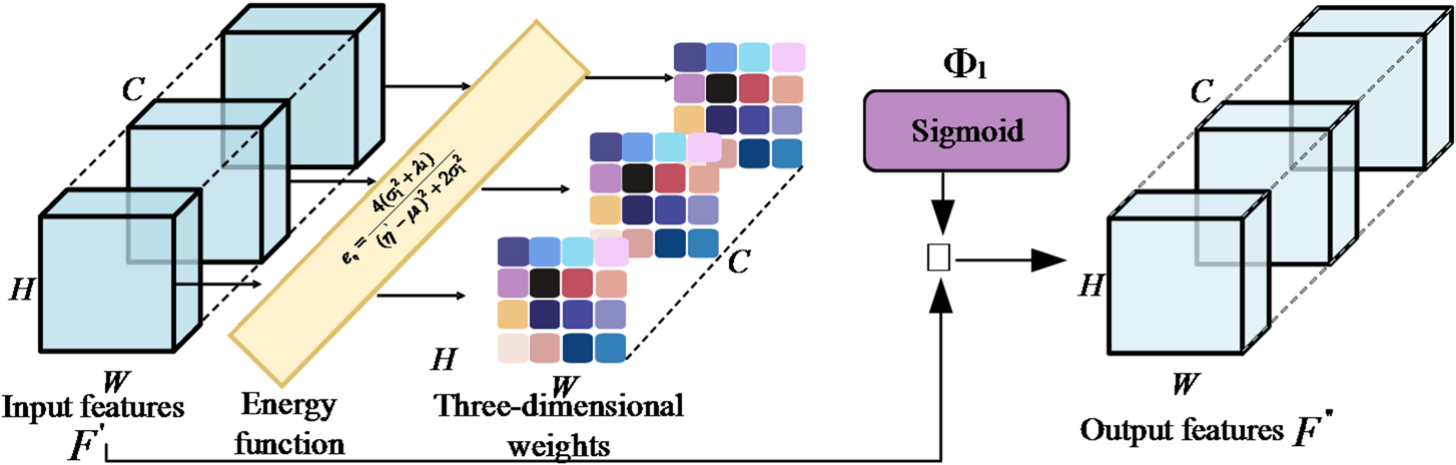
ISimAM module.

Following the channel-wise weighting of the feature map $F \in \mathbb{R}^{{C \times H \times W}}$ within the DPFD-ACAM module, the recalibrated output ${F^\prime}$ is obtained. To further enhance spatial sensitivity toward subtle thermodynamic anomalies, the Improved SimAM (ISimAM) is introduced. Unlike conventional channel attention, which often masks localised fault signatures through global pooling^39^. ISimAM independently evaluates each neuron to isolate microscopic perturbations from dominant background noise. As illustrated in Fig. 3, ISimAM is a parameterless 3D architecture specifically re-engineered for engine health monitoring. Inspired by the spatial inhibition theory, it calculates an importance weight for each neuron, enabling fine-grained calibration of transient thermal deviations. The mechanism comprises three refined stages: adaptive energy function construction, sensitivity-based importance evaluation, and feature recalibration. By introducing a fluctuation penalty factor into the energy function, ISimAM can effectively amplify low-magnitude diagnostic signals, ensuring that even weak faults are accurately captured within the complex spatio-temporal feature manifold. Initially, ISimAM defines an energy function to identify highly informative neurons, which typically exhibit firing patterns distinct from those of their neighbours. For any target neuron ${\eta^\prime}$, the minimum energy $e_\eta ^\prime$ is derived using a closed-form solution, as expressed in Eq. (9).

9 $${e_{_\eta ^\prime}} = {{4(\sigma _1^2 + {\lambda _1})} \mathord{\left/{\vphantom {{4(\sigma _1^2 + {\lambda _1})} {{{({\eta ^\prime} - {\mu _1})}^2} + 2\sigma _1^2}}} \right.\kern-\nulldelimiterspace} {{{({\eta ^\prime} - {\mu _1})}^2} + 2\sigma _1^2}}$$

where ${\lambda _1}$ represents the weak-signal amplification factor, ensuring the model prioritizes neurons with abnormal fluctuation patterns. ${\eta ^\prime}$ represents the value of the target neuron, while ${\mu _1}$ and $\sigma _1^2$ denote the mean and variance of all other neurons within the same channel, respectively. The term $\sigma _1^2$ is a small regularisation hyperparameter introduced to ensure numerical stability by preventing division by zero. A lower energy value ${e_\eta }$ indicates that the target neuron $\eta ^\prime$ It is highly differentiated from its spatial neighbourhood, signifying greater information density and, consequently, higher importance. Building on this, a feature recalibration strategy is implemented. Given that neuronal importance is inversely proportional to the energy level, the attention weight for each neuron is derived by applying a Sigmoid activation to the reciprocal of the energy ${e_i}$. Finally, the resulting 3D attention map is applied to the input feature map ${F^\prime}$ via element-wise multiplication, yielding the final refined output ${F^{\prime\prime}}$, as expressed in Eq. (10).

10 $${F^{\prime\prime}} = {\Phi _1}({1 \mathord{\left/{\vphantom {1 {{e^{\prime\prime}}}}} \right.\kern-\nulldelimiterspace} {{e^{\prime\prime}}}}) \odot {F^\prime}$$

where ${e^{\prime\prime}}$ represents the energy tensor encompassing the values ${e_{_\eta ^\prime}}$ of all constituent neurons. The term ${\Phi _1}$ denotes the Sigmoid activation function, which serves to normalise the energy reciprocals of the energy into a probability-like importance map. Finally, $\odot$ denotes the Hadamard product (element-wise multiplication), which integrates the 3D attention weights with the input features to produce the recalibrated output.

The proposed architecture extracts high-fidelity $C \times H \times W$ feature maps ${F^{\prime\prime}}$ by synergising the DPFD backbone with the dual attention mechanism. To balance diagnostic complexity with computational efficiency, a streamlined classifier head, comprising a Global Average Pooling (GAP) layer and a single Fully Connected (FC) layer, is utilised to generate the final diagnostic output. Specifically, the output feature map ${F^{\prime\prime}}$ undergoes global averaging via the GAP layer. By compressing the spatial dimensions $H \times W$ of each feature channel into a $1 \times 1$ single scalar, representing the mean activation intensity of that channel, the model distils the aggregate feature information into a *C*-dimensional feature vector $\ell$. For a total of *k*($k = 1, \cdots ,C$) channels, the corresponding global feature vector ${\ell _k}$ is formulated as follows in Eq. (11).

11 $${\ell _k} = {{(1} \mathord{\left/{\vphantom {{(1} {H \times W)}}} \right.\kern-\nulldelimiterspace} {H \times W)}}\sum\limits_{i = 1}^H {\sum\limits_{j = 1}^W {F_k^{\prime\prime}(i,j)} }$$

In this formula, $F_k^{\prime\prime}(i,j)$ represents the activation response of the *k*-th feature channel map at the spatial coordinates (*i*, *j*). Subsequently, the *C*-dimensional feature vector $\ell$ produced by the GAP layer is fed into a fully connected (linear) layer to compute the prediction scores for each target class. For a diagnostic task encompassing *U* distinct categories, the output logits vector $I \in {\mathbb{R}^u}$ is derived using Eq. (12).

12 $$I = {\tau _7} \cdot \ell + {b_3}$$

In this formulation, ${\tau _7} \in {^{K \times C}}$ represents the weight matrix of the fully connected layer, and ${b_3} \in {\mathbb{R}^K}$ denotes the corresponding bias vector. To generate a final diagnostic interpretation, the raw logits scores *I* are transformed into a probability distribution vector *p* using the Softmax activation function. For the *q*-th category, the final prediction probability ${p_q}$ is defined as follows in Eq. (13). Ultimately, the DPFD-JCSAM model employs a maximum likelihood criterion, selecting the class with the highest probability as the final diagnostic result.

13 $${p_q} = \mathrm{Softmax}(I){}_q = {{\exp ({I_q})} \mathord{\left/{\vphantom {{\exp ({I_q})} {\sum\limits_{r = 1}^u {\exp ({I_r})} }}} \right.\kern-\nulldelimiterspace} {\sum\limits_{r = 1}^u {\exp ({I_r})} }}$$

### Co-evolutionary optimization based on CRIME

The performance of the *DPFD-JCSAM* model depends on several critical hyperparameters. Specifically, it simultaneously optimizes the global training hyperparameters (i.e., initial learning rate and batch size), the structural capacity of the DPFD module (i.e., convolutional channel base), and the feature recalibration bottleneck of the JCSAM module (i.e., channel attention reduction ratio). Traditional optimisation strategies, such as grid search or random search, are computationally expensive and often fail to identify the global optimum in complex, non-convex parameter spaces. To address these inefficiencies, the Rime optimization algorithm (RIME) optimisation algorithm was recently introduced. As a novel metaheuristic inspired by the formation of rime and ice in nature, RIME offers a more robust alternative to conventional search methods^40^. The fundamental mechanisms of standard RIME, including agent position updates, global exploration, and local exploitation, are as follows:

14 $$\left\{ \begin{array}{l}\text{Global exploration}:\xi (t + 1) = {\xi _{\mathrm{best}}}(t) + {R_1} \cdot ({\xi _{{r_1}}}(t) - {\xi _{{r_2}}}(t))\\\text{Local exploitation}:{\xi _s}(t + 1) = {\xi _s}(t) + {R_2} \cdot ({\xi _{\mathrm{best}}}(t) - {\xi _s}(t))\end{array} \right.$$

where $\xi (t)$ denotes the agent position at iteration *t*, ${\xi _{\mathrm{best}}}$ represents the current global optimal solution, and ${\xi _s}(t)$ signifies the current position of the *s*-th agent. The term *R*_1_ is a stochastic factor, while ${\xi _{{r_1}}}$ and ${\xi _{{r_{_2}}}}$ represent two agents randomly selected from the population. The variable *R*_2_ represents a random step size drawn from a uniform distribution. To address the susceptibility of standard RIME to local optima and the imbalance between exploration and exploitation in high-dimensional spaces, the SRIME variant introduced the Lévy flight strategy and an Elite Survival Selection mechanism^41^. However, despite these enhancements, Strengthened RIME (SRIME) essentially relies on a set of compromised rules to guide the entire population, which often fails to achieve an optimal state across different evolutionary stages. To identify the optimal hyperparameter configuration with greater efficiency and robustness, this paper proposes an advanced metaheuristic: the Cooperative Rime Information Migration Evolutionary (CRIME). The fundamental concept of CRIME involves the functional decomposition of the search population into two parallel, specialised sub-populations linked by an information exchange mechanism. Specifically, the Exploration Swarm is dedicated to conducting broad, global searches. By replacing the standard RIME soft-rime process with a Lévy-flight-based exploration mechanism, this swarm utilises a random walk characterised by alternating long and short steps. This approach facilitates a comprehensive search of the solution space, mitigating the risk of premature convergence to local optima. The position update formulation for the Exploration Swarm is as follows:

15 $$\xi (t + 1) = {\xi _s}(t) + \chi \odot {\rm{L}}evy(\vartheta )$$

In this formulation, ${\rm{L}}evy(\vartheta )$ represents the stochastic step of a Lévy flight, characterised by a heavy-tailed distribution that enables long-range jumps to escape local entrapment. Concurrently, ${\xi _s}(t)$ denotes the current position of the *s*th agent within the Exploitation Swarm. This sub-population is dedicated to high-precision local search, employing a chaotic-map-based search strategy. To ensure refined, non-redundant exploration of promising regions already identified, a classical Logistic map is used to generate a perturbed chaotic sequence, ${l_k}$. This sequence exhibits superior ergodicity and sensitivity to initial conditions compared with standard random walk strategies, facilitating more efficient convergence towards the global optimum. The Logistic map is defined as follows in Eq. (16).

16 $$\left\{ \begin{array}{l}{l_{k + 1}} = \varpi \cdot {l_k} \cdot (1 - {l_k}),{l_k} \in (0,1)\\\xi (t + 1) = {\xi _s}(t) + \gamma \cdot ({l_k} - 0.5)\end{array} \right.$$

where $\gamma$ denotes the control coefficient governing the disturbance range, while $\varpi$ regulates the spread of the perturbation sequence. Using the chaotic sequence ${l_k}$, the operator $\xi (t + 1)$ iteratively fine-tunes the current global best solution to generate refined candidate solutions. To bridge the functional gap between the two specialised swarms, the algorithm incorporates an Information Migration mechanism to facilitate co-evolution. This strategy ensures a dynamic and efficient balance between global exploration and local exploitation throughout the evolutionary trajectory. By implementing the CRIME algorithm, the model identifies a hyperparameter configuration that approximates the global optimum. This optimisation significantly amplifies the performance potential of the front-end dynamic feature extraction architecture, establishing a rigorous foundation for the high diagnostic accuracy and operational robustness of the proposed framework.

### Fusion-CAM visualisation module

To overcome the inherent opacity that limits fault diagnostic reliability, a Fusion Class Activation Mapping (Fusion-CAM) visualisation module is introduced to fundamentally break the “black-box” nature of the CDAF’s multi-source feature fusion. This comprehensive interpretability scheme ensures highly reliable diagnostics by quantitatively mapping local thermal parameter responses to the global engine health status. To bridge this gap, a post hoc explainability framework is employed. Gradient-weighted Class Activation Mapping (Grad-CAM) is utilised to generate visual heatmaps that reveal the specific regions of the thermal parameter maps prioritised by the model during a particular diagnostic prediction^42^. However, a notable limitation of standard Grad-CAM is its reliance on the Global Average Pooling (GAP) operation. By aggregating spatial gradient information into a single scalar, GAP assumes uniform importance across the spatial domain, thereby neglecting the heterogeneous contributions of distinct spatial coordinates to the final classification. This often results in a coarse localisation map that lacks fine-grained resolution. The mathematical formulation for standard Grad-CAM is as follows.

17 $$L_{Grad - CAM}^u = \mathrm{ReLU(}\sum\limits_k {(\frac{1}{{H \cdot W}}\sum\limits_i^H {\sum\limits_j^W {{{(\partial {y^u}} \mathord{\left/{\vphantom {{(\partial {y^u}} {\partial {\Gamma }_{ij}^k)}}} \right.\kern-\nulldelimiterspace} {\partial {\Gamma }_{ij}^k)}}} } ) \cdot {{\Gamma }^k}} )$$

In this formulation, *H* and *W* denote the height and width of the feature map, respectively. $L_{Grad - CAM}^u$ represents the weighted summation across all feature channels, where the specific contribution to state *u* is isolated using a ReLU function. ${\Gamma }_{ij}^k$ refers to the pixel intensity at coordinates (*i*, *j*) of the *k*-th channel. While optimised variants like Grad-CAM++^30^ and Score-CAM^31^ improve upon the standard Grad-CAM^29^ by refining gradient and channel weighting, they remain linear, single-attribution processes confined to the spatial dimension. Since the proposed framework collaboratively fuses generalised multi-source features from different branches at the fusion layer, it is necessary to quantitatively assess the individual contribution of each branch to the final diagnostic decision. To address this, we propose Fusion Class Activation Mapping (Fusion-CAM). This method transcends the limitations of traditional spatial visualisation by jointly quantifying the dual contributions of spatial location and feature channels. This approach clearly reveals the deep fusion logic governing the model’s focus areas and ensures the physical traceability of specific diagnostic paths. Specifically, the target layer for Fusion-CAM is the final convolutional layer of the fusion stage. Unlike traditional gradient-based methods, Fusion-CAM calculates weights based on the final decision score relative to the fusion layer’s feature maps, which encompass a total of 2*c* channels. Following Global Average Pooling, 2*c* distinct channel weights are derived. These weights $w_{_{2c}}^\prime$ and their corresponding feature maps ${f_{2c}}$ are then partitioned into two discrete groups according to their original path: the first *c* channels correspond to the trend feature maps, while the remaining *c* channels correspond to the mean feature maps. By performing a weighted summation for each group independently, two distinct heatmaps are generated.

The process begins by passing the dual-source input samples through the model in a full forward pass to obtain the prediction score *Y*_*o*_ for the target category *o*. During this pass, the activation maps of the fusion layer, with dimensions (2*c*, *H*, *W*), are extracted and stored. Subsequently, the algorithm executes backpropagation to compute the gradients and weights. Following the conventional gradient-weighting strategy, we derive the score $Y_o^u$ for category *u* relative to the gradient $\partial Y_o^u/\partial {f_k}$ of the *k*-th channel feature map ${f_k}$ within the fusion layer. The resulting gradient tensor maintains the dimensions (2*c*, *H*, *W*). To determine the significance of individual feature branches, global average pooling is applied across the spatial dimensions (*H*, *W*) for each of the 2*c* gradient channels. This operation yields the importance weight $w_{k}^\prime$ for the *k*-th channel, as defined in Eq. (18).

18 $$w_{_k}^\prime = \frac{1}{{H \cdot W}}\sum\limits_i^H {\sum\limits_j^W {{{(\partial Y_o^u} \mathord{\left/{\vphantom {{(\partial Y_o^u} {\partial f_{ij}^k)}}} \right.\kern-\nulldelimiterspace} {\partial f_{ij}^k)}}} } ,k \in [1,2c]$$

Building on the derived weights, the attribution analysis is performed via a linear weighted combination of the feature channels. To isolate the influence of the individual diagnostic streams, the fusion layer is partitioned into two discrete branches, each corresponding to a distinct data source. The first branch, corresponding to the trend-based features, utilises the first *c* elements of the weight vector $w_{k}^\prime$($w_{1}^\prime,w_2^\prime, \cdots ,w_{c}^\prime$) and the first *c* channels of the feature map ${f_{{\rm{path}}\mathrm{1},k}}$. Conversely, the second branch, representing the mean-based features, utilises the remaining *c* elements of the weight vector $w_{k - c}^\prime$($w_{{c + 1}}^\prime,w_{c + 2}^\prime, \cdots ,w_{{2_c}}^\prime$) and the corresponding final *c* channels of the feature map ${f_{path 2,k-c}}$. By performing independent weighted summations for each branch, the model generates distinct heatmaps for both the dynamic trends and static means. This dual-branch visualisation enables a granular assessment of which physical indicators, evolutionary patterns, or steady-state magnitudes the model prioritises for a given diagnostic outcome. The source-specific heatmap generation is governed by Eq. (19).

19 $$\left\{ \begin{array}{l}{L_{\mathrm{fusion\_cam1}}} = {\mathrm{ReLU}(}\sum\limits_k^c {w_k^\prime \cdot {f_{{\rm{path}}{1},k}}} {)}\\{L_{\mathrm{fusion\_cam2}}} = \mathrm{ReLU(}\sum\limits_{k = c + 1}^{2c} {w_{k - c}^\prime \cdot {f_{{\rm{path}}{2},k - c}}} {)}\end{array} \right.$$

To facilitate intuitive interpretation, the resulting activation maps are upsampled and superimposed on the original input data for visualisation. Specifically, the source-specific heatmaps, *L*_fusion_cam1_ and *L*_fusion_cam2_, are bilinearly interpolated to match the original dimensions of feature branch 1 (Trend) and feature branch 2 (Mean), respectively. By overlaying these heatmaps on the raw thermal parameter grids, the model provides a transparent visualisation of the specific regions driving the diagnostic decision. Furthermore, this method enables the expansion and inspection of individual channel heatmaps, allowing engineers to trace diagnostic evidence back to specific sensors or time windows. This high-resolution localisation bridges the gap between deep learning abstractions and the physical reality of diesel engine fault evolution. Crucially, beyond passive visualisation, the generated Fusion-CAM heatmaps are quantified into a set of diagnostic reliability weights. These weights serve as a knowledge-based feedback mechanism, enabling the framework to distinguish between fault-induced deviations and stochastic noise. By mapping these importance scores back to the input space, the system can adaptively refine its focus on high-contribution thermal parameters, transitioning from simple interpretation to an active, self-correcting diagnostic process. This ensures that the black-box decisions are both verifiable and physically grounded.

### Explainable weak thermal fault diagnosis based on CDAF

**Fig. 4:**
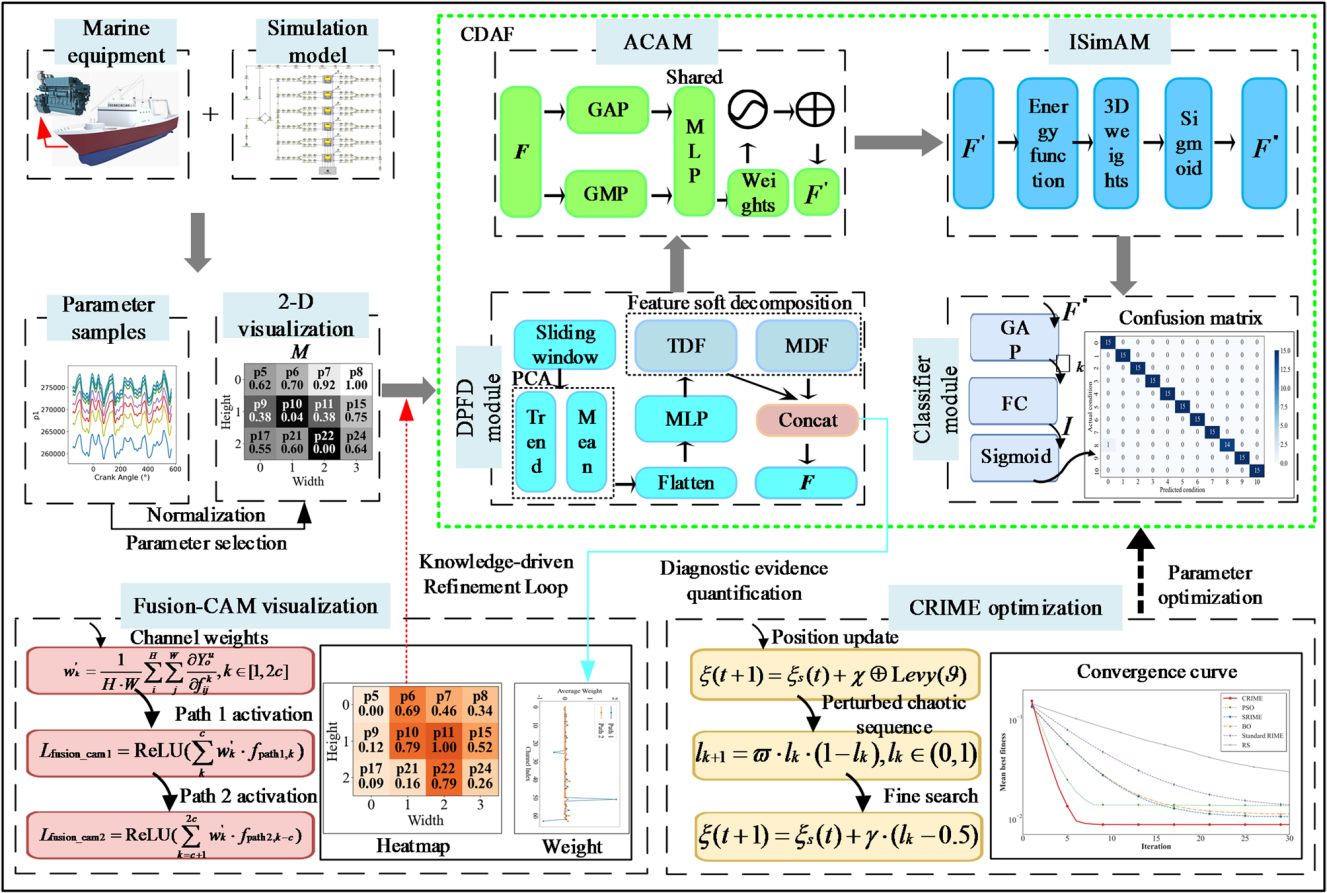
The flowchart of CDAF.

To address the challenge of extracting weak drift features under strong noise conditions, as well as the limitations of existing methods in quantifying the independent decision contribution of each branch within a generalised multi-source fusion layer, this study proposes an explainable CDAF for weak thermal fault diagnosis. The flowchart of CDAF is shown in Fig. 4. The framework leverages the DPFD-JCSAM to precisely isolate subtle drift characteristics and capture the complex coupling relationships between high-dimensional nonlinear features and nascent thermal fault states. Concurrently, the CRIME is employed to navigate the vast hyperparameter space, identifying the global optimal configuration to ensure superior classification performance. Finally, based on the diagnostic outcomes, the Fusion-CAM visualisation method is implemented to quantify the decision contribution of different branches within the multi-source feature fusion layer, thereby enabling physically explainable traceability of the model’s decision logic. The comprehensive architecture of the CDAF is illustrated in Fig. 4, with the specific procedural steps detailed below.

1. Hybrid dataset construction and preprocessing. A comprehensive weak-fault dataset is constructed by integrating experimental diesel engine bench test data with one-dimensional thermodynamic simulation data, creating a hybrid virtual-real sample set. key thermal parameters are then screened and normalised using correlation analysis and mutual information metrics. This step eliminates dimensional inconsistencies and ensures high-quality input for robust feature extraction.
2. Co-evolutionary global optimisation and decoupled feature extraction. During the model’s training phase, the entire CDAF model is dynamically optimized by the CRIME algorithm. Driven by this co-evolutionary engine, the preprocessed data is fed into a physically-informed Dual-Path Feature Decoupling (DPFD) module. Acting as a trend-mean dual-guided filter, the DPFD effectively isolates incipient thermodynamic degradation drifts from high-intensity, stochastic sea-state noise, producing highly robust dual-path feature representations.
3. Transparent feature fusion and attention weighting. To process the decoupled features, a Joint Channel-Spatial Attention Mechanism (JCSAM) is designed for transparent feature fusion. The ACA module and I-SimAM network work synergistically to adaptively recalibrate weights across both channel and spatial dimensions. This dual-stage refinement successfully delineates the fuzzy boundaries of highly intertwined health states in the high-dimensional feature space, ensuring the reconstructed manifold is physically discriminative.
4. Fault recognition and classification. The highly discriminative feature representations are flattened and fed into fully connected layers. A Softmax classifier is then employed to calculate the probability distribution across different health states, ultimately producing the deterministic weak fault diagnostic results for the marine diesel engine.
5. Comprehensive interpretability evaluation and feedback. During the inference evaluation, a comprehensive interpretability evaluation scheme based on Fusion-CAM is established. It extracts diagnostic evidence fingerprints by computing the normalised gradient flow through the feature fusion layer. This step fundamentally breaks the “black-box” nature of dynamic multi-source parameter fusion by quantitatively mapping local thermal parameter responses to the global engine health status, ensuring highly reliable diagnostics and providing engineers with traceable physical evidence.

## Experimental study

### Data description

**Fig. 5:**
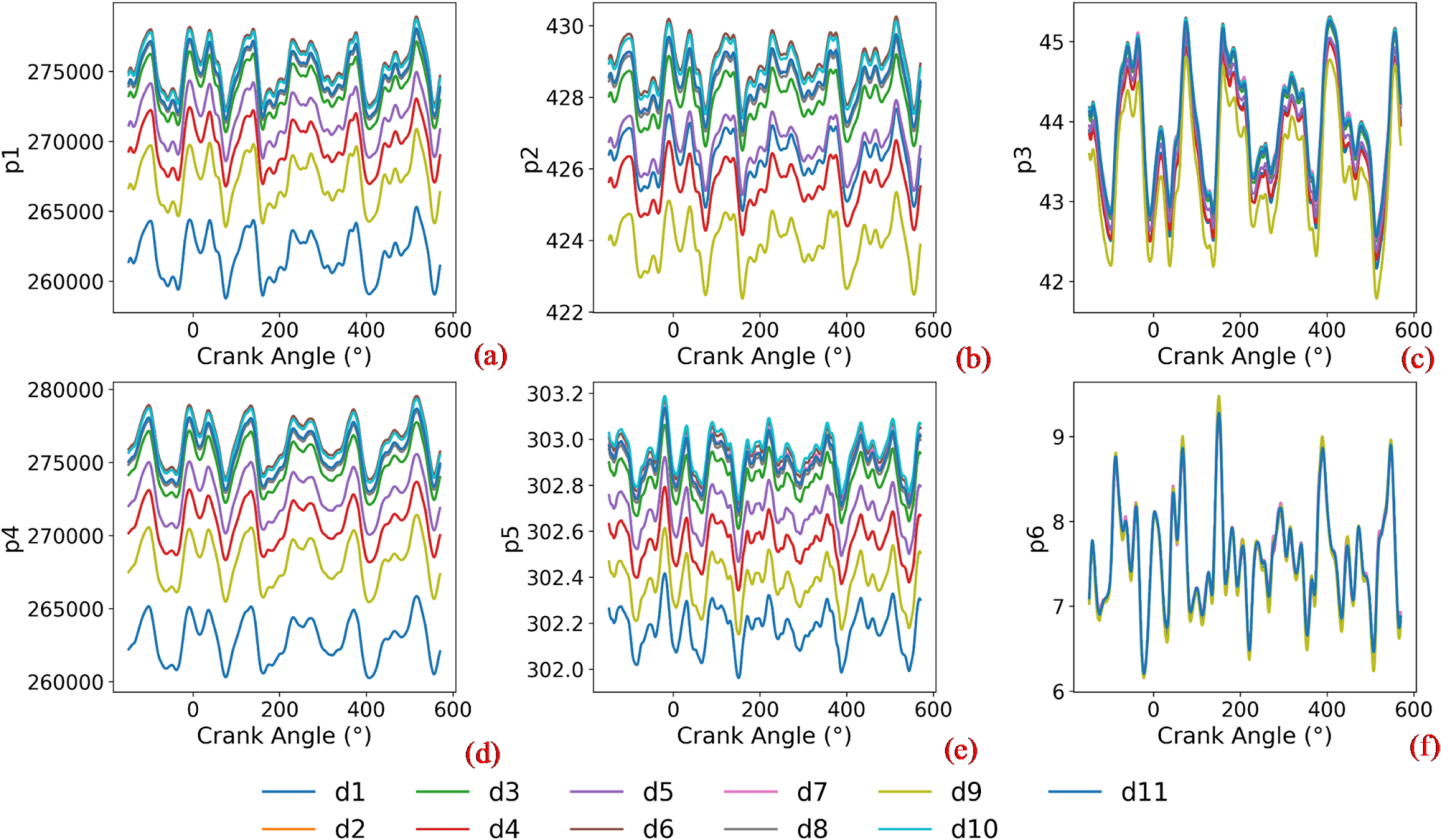
Raw data curves under different health states. Variation of partial thermal parameters with crankshaft angle. (**a**) Pressure of intake gas after turbocharger (p1), (**b**) Temperature of intake gas after turbocharger (p2), (**c**) Velocity of intake gas after turbocharger (p3), (**d**) Charge air pressure after intercooler (p4), (**e**) Charge air temperature after intercooler (p5), (**f**) Intercooler velocity (p6).

Empirical data were collected from the operational monitoring system of a vessel equipped with an L6160ZLCZ-89 marine diesel engine in the Liushawan waters of Leizhou City, Guangdong, China. Guided by technical manuals, industrial expertise, and recent literature on thermal parameter studies^4,16,43,44^, an initial screening was performed to eliminate parameters with low correlation with engine health states. The final selection of monitored parameters and diagnostic fault states is detailed in Tables 1 and 2, respectively. The characteristic profiles of representative thermal parameters with respect to the crankshaft angle are illustrated in Fig. 5. The raw datasets underwent a rigorous preprocessing pipeline, including the removal of redundant or blank records and the imputation of missing values. Furthermore, a sliding window algorithm was employed to detect and correct statistical outliers. A significant challenge in this study was integrating heterogeneous sequences: thermal parameters are indexed by the crank angle, typically ranging from 0° to 720° CA, whereas performance parameters are recorded as time-series data. To achieve temporal synchronisation, a crank-to-time mapping strategy was implemented. A single full thermodynamic cycle (i.e., 720° CA) corresponds to approximately 0.12 s under rated operating conditions; therefore, the performance parameters were resampled within a 0.12-s window. These sequences were then aligned with the sequential thermal parameter cycles in a stacked configuration, thereby unifying the heterogeneous data into a consistent temporal framework. Due to the scarcity of actual weak fault samples in practical maritime operations, the preprocessed empirical data was utilized to calibrate a 1D thermodynamic model for generating comprehensive and balanced fault conditions. To ensure a robust foundation for this virtual-real hybrid approach, the empirical dataset was filtered to harmonize with the parameter types supported by the 1D thermodynamic simulation model, ensuring a robust foundation for subsequent validation of the virtual-real hybrid model. Crucially, the fidelity of the underlying 1D thermodynamic simulation has been previously verified, demonstrating a relative error of less than 5% across critical engine performance parameters^33^.

**Table 1:** Definitions and descriptions of marine diesel engine thermal parameters.

Parameters	Unit	Label	Parameters	Unit	Label
Pressure of intake gas after the turbocharger	Pa	p1	Pressure of exhaust gas before the turbocharger	Pa	p13
Temperature of intake gas after turbocharger	K	p2	Temperature of exhaust gas after turbocharger	K	p14
Velocity of intake gas after turbocharger	m s^−1^	p3	Pressure of exhaust gas after the turbocharger	Pa	p15
Charge air pressure after intercooler	K	p4	Velocity of exhaust gas after turbocharger	m s^−1^	p16
Charge air temperature after intercooler	K	p5	Temperature of intake manifold	K	p17
Intercooler velocity	m s^−1^	p6	Pressure of intake manifold	Pa	p18
Maximum pressure during combustion	Pa	p7	Temperature of exhaust manifold	K	p19
Maximum temperature during combustion	K	p8	Pressure of exhaust manifold	Pa	p20
Brake power	kW	p9	Velocity of exhaust manifold	m s^−1^	p21
Brake specific fuel consumption	g kW^−1^ h m s^−1^	p10	Temperature of exhaust	K	p22
Brake mean effective pressure	Pa	p11	Pressure of exhaust	Pa	p23
Temperature of exhaust gas before turbocharger	K	p12	Flow velocity of exhaust	m s^−1^	p24

**Table 2:** Definitions and descriptions of marine diesel engine fault types.

Fault type	Label	Details	Fault type	Label	Details
Compressor blade fouling	d1	Flow multiplier: 0.98, efficiency Multiplier: 0.98	Early exhaust valve closure	d7	Advance exhaust valve closing by 1.0–2.0°CA
Cooler air-side blockage	d2	Increase the pressure loss coefficient by 10–20%	Intake valve leakage	d8	Equivalent leakage area 0.1–0.5 mm²
Injector timing advance	d3	Advance injection starts by 0.5–1°CA	Turbine blade fouling	d9	Flow Multiplier:0.98, efficiency multiplier: 0.98
Injector nozzle clogging	d4	Reduce single-cycle injection by 1–2%	Compression ratio reduction	d10	Compression ratio reduced from 14.5:1 to 14.2:1 or 14.4:1
Uneven single-cylinder fuel supply	d5	Adjust the injection of a single cylinder by ± 3%, others unchanged	Normal condition	d11	
Ignition delay	d6	Delay combustion start by 0.5–1°CA			

### Experimental setting

To evaluate the diagnostic framework, the inherent heterogeneity in the scales of raw thermal parameters poses a risk of weight assignment bias during model training. To mitigate this skewness, a min-max normalization strategy is applied to map all features onto a standardized scale, enforcing equitable parameter contributions across the deep learning framework. Following normalization, samples are randomly partitioned into training, validation, and test sets in proportions of 70%, 15%, and 15%. Model training was conducted using the Adam optimiser over 50 epochs, with an initial learning rate of 3e−3 and a mini-batch size of 32. To ensure experimental reproducibility and facilitate comparative analysis, a global random seed was fixed (Random Seed = 42). Diagnostic efficacy was quantified using a comprehensive suite of metrics, including accuracy, precision, recall, and the F1-score. The specific structural parameters for the core modules were configured as follows:

1. DPFD module: The reconstruction weight coefficient *α* was set to 0.5, and the feature dimensionality reduction ratio *γ* was set to 4.
2. ACAM module: A convolution kernel size of 3 × 3 was utilised within the shared MLP, and the channel reduction ratio was set to 16 to balance representational power with computational efficiency.
3. ISimAM module: Consistent with its parameter-free design, this lightweight module adaptively derives neuron-level weights directly from input feature statistics without the need for additional learnable parameters.

**Fig. 6:**
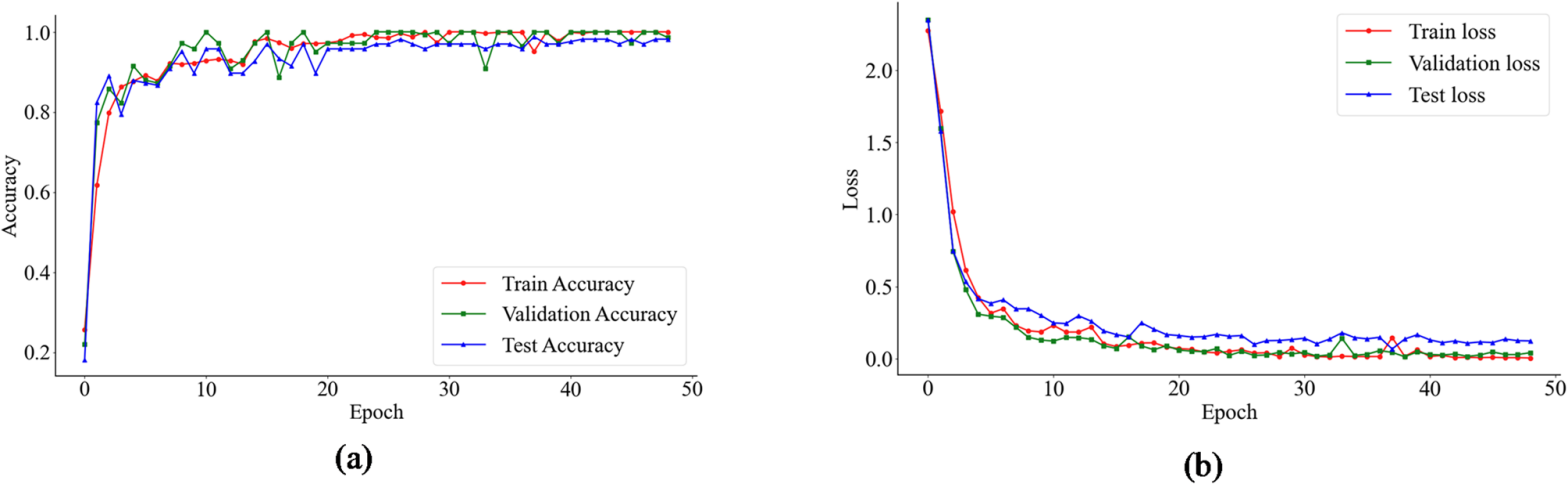
Accuracy and loss curves of the model on the training, validation, and test sets. (**a**) Accuracy curve, (**b**) loss curve.

Figure 6 shows the accuracy and loss trajectories of the proposed CDAF across the training, validation, and test datasets as a function of training epochs. As depicted in Fig. 6a, the model exhibits an accelerated learning phase during the initial stages of training, with accuracy across all three datasets rising sharply from approximately 20% to over 95%. This rapid ascent indicates that the architecture is highly effective at identifying and capturing discriminative features from the hybrid virtual-real dataset. Beyond the 15th epoch, the accuracy curves enter a steady-state convergence phase, gradually saturating near the optimal performance level. Complementing the accuracy trends, Fig. 6b shows the loss function behaviour. The model’s loss undergoes a near-vertical decline during early training, plummeting from an initial value exceeding 2.0 to below 0.2. Subsequently, the loss continues a marginal, asymptotic descent, eventually stabilising at a level approaching zero. This consistent reduction, without significant divergence between the training and validation sets, confirms that the model has reached a fully optimised state without overfitting. In summary, the convergence characteristics demonstrate that the proposed framework is robust, characterised by rapid convergence, high diagnostic precision, and superior generalisation across heterogeneous data sources.

### Architecture and comparative methods

The proposed CDAF comprises four functional pillars: feature extraction, classification, global parameter optimisation, and post hoc explainability analysis. Using generalised multi-source thermal parameters as input, the framework first employs the DPFD module to achieve deep decoupling of latent features from background noise, thereby constructing a high-fidelity input space in which fault-related signals remain distinguishable under intense background noise. Subsequently, the dual attention mechanism, comprising the ACAM and the ISimAM, is integrated to perform multi-dimensional feature focusing and dynamic recalibration of high-discriminative characteristics. Building on this refined feature set, the CRIME is executed to navigate the global parameter space and identify the optimal hyperparameter configuration. Finally, the extracted high-dimensional features are mapped to diagnostic labels via a fully connected classification layer.

To rigorously evaluate the diagnostic efficacy of the proposed CDAF, its performance was benchmarked against four representative state-of-the-art methods widely used in marine diesel engine fault diagnosis: CNN-LSTM^12^, WCNN-LSTM^14^, CNN-GRU-KAN^16^, and TSRF^17^. These models represent a diverse range of architectural strategies, including hybrid temporal-spatial networks and KAN integrations. Furthermore, to dissect the specific contributions of each innovative component, an incremental ablation study was conducted. Starting from a standard Convolutional Neural Network (CNN) baseline, the DPFD (feature decoupling), ACAM (channel attention), ISimAM (spatial attention), and CRIME (global optimisation) modules were introduced sequentially. This systematic verification process quantifies the marginal performance improvements provided by each module, ensuring that the final integrated framework is both efficient and mathematically justified.

### Discussion

#### Ablation study

**Fig. 7:**
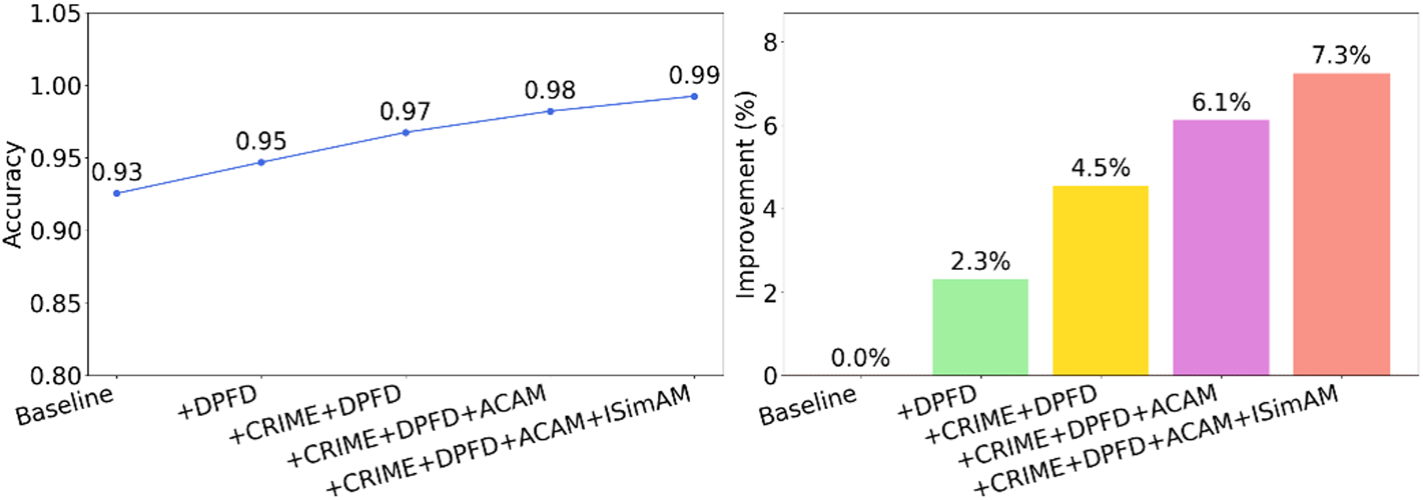
Accuracy curves of different modules and bar charts of performance improvement relative to the baseline model.

To systematically evaluate the individual contribution of each core module within the CDAF, a rigorous ablation study was conducted. The study defines four configurations: Ablation 1 (CRIME-DPFD-ACAM without ISimAM), Ablation 2 (CRIME-DPFD, without any attention mechanisms), Ablation 3 (Standard DPFD), and Ablation 4 (Baseline CNN). This incremental approach quantifies performance gains as the DPFD architecture, CRIME optimiser, and dual attention mechanisms, namely the ACAM and ISimAM, are sequentially integrated. The performance metrics, accuracy, precision, recall, and F1-score, are summarised in Table 3. The experimental results demonstrate that each introduced module makes a positive and quantifiable contribution to the framework’s overall diagnostic capability. As illustrated by the accuracy trajectories and modular gain analysis in Fig. 7, several key insights emerge:

1. The role of DPFD: Replacing the baseline CNN with the DPFD architecture produced the most significant initial boost, with diagnostic accuracy increasing by 2.3%. This underscores the importance of decoupling slow-varying trends and suppressing noise for effective feature extraction.
2. The impact of CRIME optimisation: Integrating the CRIME module with DPFD produced a secondary significant performance boost, increasing accuracy by another 2.2%. This confirms that the co-evolutionary metaheuristic effectively identifies superior hyperparameter configurations that manual tuning might overlook.
3. The synergy of dual attention: The addition of the ACAM module further improved accuracy by 2.4%, while the subsequent inclusion of the ISimAM spatial attention module provided a final refined gain of 1.2%.

Combined, these results rigorously validate the rationale and advancement of the proposed model design. From the fundamental signal decoupling of the DPFD architecture to the global search capabilities of CRIME, and finally to the fine-grained optimisation of the ACAM and ISimAM dual-attention layers, each component plays an indispensable role. Together, they form a cohesive diagnostic system characterised by high precision, stability, and robustness in the presence of complex thermal fault features.

**Table 3:** Comparative performance of different modules on the self-constructed dataset.

Model/index	Accuracy (%)	Precision (%)	Recall (%)	F1-score (%)
Baseline	92.54 ± 0.62	92.31 ± 0.75	92.83 ± 0.59	92.57 ± 0.48
DPFD	94.68 ± 0.45	94.82 ± 0.51	94.59 ± 0.48	94.70 ± 0.35
CRIME-DPFD	96.75 ± 0.38	96.88 ± 0.42	96.65 ± 0.41	96.76 ± 0.29
CRIME-DPFD-ACAM	98.21 ± 0.25	98.15 ± 0.33	98.33 ± 0.28	98.24 ± 0.19
CRIME-DPFD-ACAM-ISimAM	99.25 ± 0.23	99.29 ± 0.28	99.22 ± 0.26	99.63 ± 0.19

#### Model evaluation

To evaluate the diagnostic efficacy of the proposed CDAF, its performance was benchmarked against a diverse array of established and cutting-edge diagnostic architectures. The comparative suite includes traditional ensemble learning (TSRF), hybrid spatiotemporal deep learning models (CNN-LSTM and WCNN-LSTM), and the recently introduced CNN-GRU-KAN. The experimental results, summarised in Table 4, demonstrate that the proposed method achieves superior diagnostic performance across all categories, with an accuracy of 99.71%, a precision of 99.75%, a recall of 99.68%, and an F1-score of 99.71%. The framework significantly outperforms the TSRF baseline, which achieves an accuracy of 93.15%, as well as hybrid models such as WCNN–LSTM, which achieves an accuracy of 96.52%. This substantial superiority is primarily attributed to the distinct feature decoupling mechanism of DPFD-JCSAM. Unlike standard hybrid models that are susceptible to noise interference, the Trend-Mean dual-guided strategy effectively filters out high-frequency noise while preserving the integrity of weak fault signatures, enabling more precise feature extraction. While the variant configurations (CRIME-MDF-ACAM-ISimAM and CRIME-TDF-ACAM-ISimAM) and the CNN-GRU-KAN model represent the strongest competitors, with accuracies hovering around 98%, the proposed integrated framework maintains a clear performance lead. Specifically, compared with the second-best-performing model, CRIME-MDF-ACAM-ISimAM, which achieves an accuracy of 98.35%, the proposed method achieves a marginal accuracy improvement of 1.36% points, along with superior precision and recall. This performance gap highlights the need for the dual-frequency fusion strategy. While single-branch variants (MDF or TDF) capture only partial information, the complete framework integrates macroscopic trend evolution with microscopic fluctuation details, ensuring a more comprehensive and discriminative feature representation. Beyond the mean performance values, the standard deviation of the metrics provides critical insight into model robustness. The proposed framework exhibits the lowest variance across all performance indices, for example, achieving an F1-score of 99.71% with a variance of 0.15%. This minimal dispersion indicates that the model not only delivers the highest average diagnostic accuracy but also ensures the most stable and reliable predictions. Such exceptional stability is physically rooted in the CRIME algorithm’s cooperative optimisation strategy. By leveraging the information migration mechanism to prevent the hyperparameter search from trapping in local optima, CRIME ensures that the network converges to a globally optimal state across different trials. Such consistency across repeated experimental trials confirms the framework’s suitability for diagnosing subtle thermal faults in marine diesel engines, where reliability is as vital as precision.

**Table 4:** Comparison of the diagnostic performance of different methods.

Model	Accuracy (%)	Precision (%)	Recall (%)	F1-score (%)
CRIME-MDF-ACAM-ISimAM	98.35 ± 0.28	98.41 ± 0.32	98.29 ± 0.30	98.35 ± 0.22
CRIME-TDF-ACAM-ISimAM	98.24 ± 0.31	98.19 ± 0.35	98.31 ± 0.29	98.25 ± 0.24
CNN-LSTM	95.71 ± 0.55	95.63 ± 0.62	95.84 ± 0.58	95.73 ± 0.49
WCNN-LSTM	96.52 ± 0.49	96.48 ± 0.53	96.61 ± 0.51	96.54 ± 0.42
TSRF	93.15 ± 0.78	93.28 ± 0.81	93.07 ± 0.85	93.17 ± 0.64
CNN-GRU-KAN	97.88 ± 0.35	97.92 ± 0.41	97.83 ± 0.39	97.87 ± 0.28
Proposed method	99.71 ± 0.19	99.75 ± 0.22	99.68 ± 0.24	99.71 ± 0.15

**Fig. 8:**
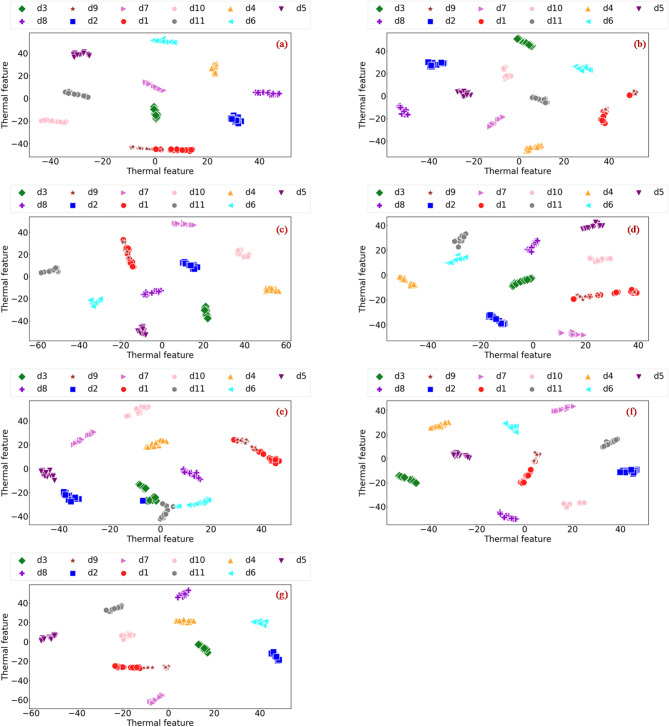
The *t*-SNE visualisation of selected sample distribution. (**a**) CRIME-MDF-ACAM-ISimAM, (**b**) CRIME-TDF-ACAM-ISimAM, (**c**) CNN-LSTM, (**d**) WCNN-LSTM, (**e**) TSRF, (**f**) CNN-GRU-KAN, (**g**) Proposed method.

To further evaluate the framework’s high-dimensional feature learning and classification capabilities from a multidimensional perspective, a series of visual analyses was conducted. Specifically, the t-Distributed Stochastic Neighbour Embedding (*t*-SNE) technique was used to project the high-dimensional latent features extracted from the model’s final layer into a two-dimensional subspace for visualisation. The comparative results are illustrated in Fig. 8. As shown in Fig. 8g, the proposed model transforms the raw data into highly discriminative feature representations. The data points corresponding to the eleven distinct categories (d1–d11) form compact clusters with well-defined boundaries. Inter-class overlap is negligible, indicating that the model has successfully learned the unique manifold structure of each fault state. This clear separation is driven by the cascaded attention mechanism within DPFD-JCSAM, which actively suppresses redundant background information and amplifies subtle, discriminative patterns specific to each thermal fault. In contrast, the *t*-SNE projections for the benchmark models, shown in Fig. 8a–f, exhibit significant confusion, particularly between classes d1 and d9, where substantial overlap and cluster intersection are observed. This suggests that without the Trend-Mean decoupling strategy, standard deep learning architectures struggle to disentangle the coupled signal variations of these similar fault types from intense background noise, leading to ambiguous decision boundaries. This intuitive visualisation confirms that the CDAF architecture can extract highly separable feature representations, providing a robust mathematical foundation for its superior diagnostic precision.

**Fig. 9:**
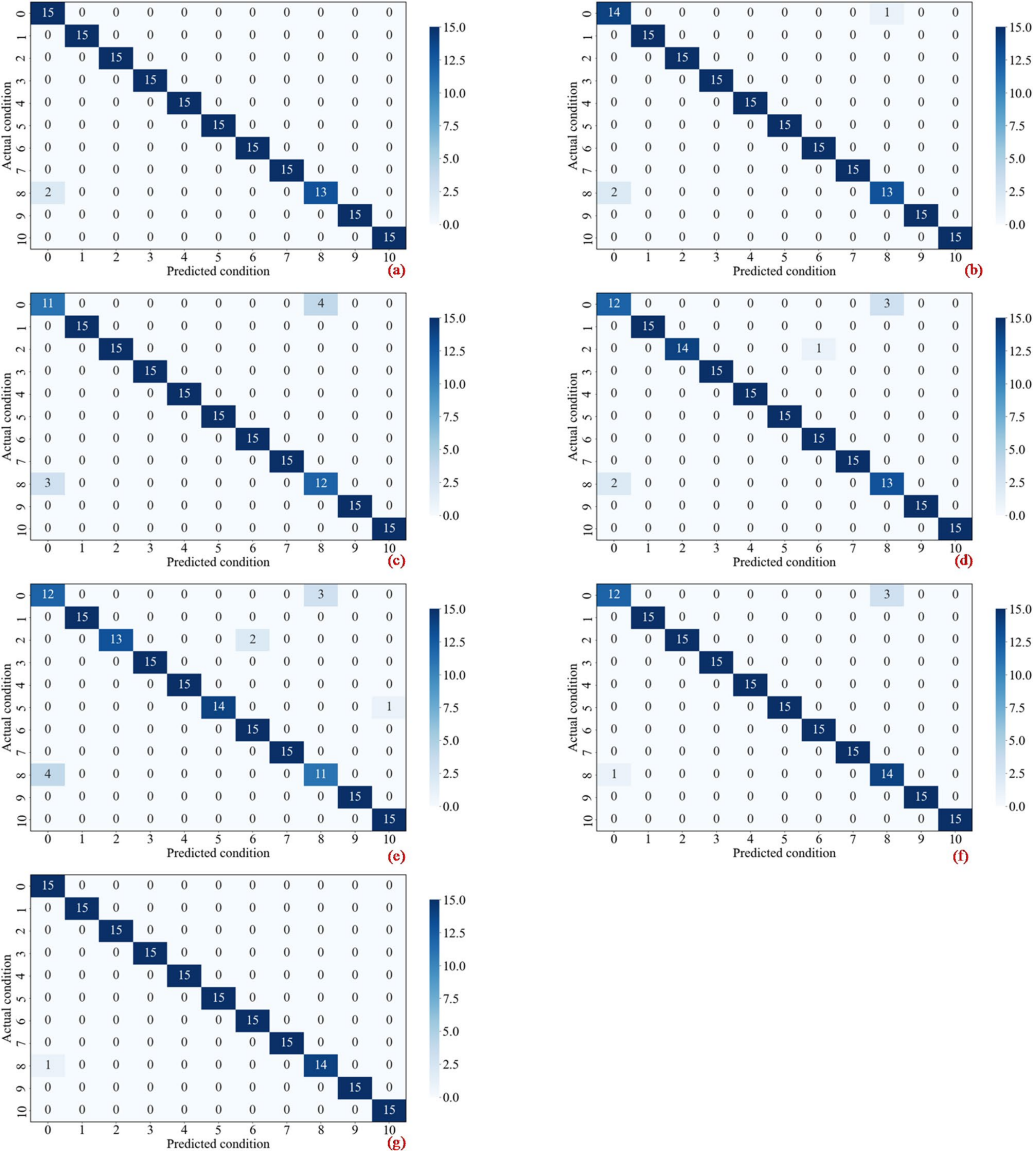
Confusion matrices for seven methods on test samples. Axes 0–10 indicate states d1–d11. (**a**) CRIME-MDF-ACAM-ISimAM, (**b**) CRIME-TDF-ACAM-ISimAM, (**c**) CNN-LSTM, (**d**) WCNN-LSTM, (**e**) TSRF, (**f**) CNN-GRU-KAN, (**g**) Proposed method.

To evaluate the proposed framework and the comparative models’ classification performance across discrete diagnostic categories, the confusion matrix for the test set is presented in Fig. 9. The results show strong diagonal dominance, with predicted labels almost perfectly aligned with ground-truth annotations across all eleven categories. This concentration on the main diagonal indicates that the model maintains exceptional sensitivity and specificity for each engine state. Off-diagonal values, representing classification errors, are negligible. Notably, the CDAF outperforms other state-of-the-art methods in distinguishing fault state d9, a category where other methods frequently struggle. While benchmark models exhibit significant inter-class confusion between states d1, d3, and d9, the proposed method eliminates these misidentifications. This reduction in confusion is attributed to the model’s ability to resolve feature aliasing among thermodynamically similar faults. By leveraging the dual-guided mechanism to process trend and noise components separately, the framework captures the subtle, unique deviation patterns of each fault that are otherwise lost in the coupled features extracted by traditional single-stream networks. The isolated and infrequent nature of the errors in the proposed framework further validates its fine-grained recognition capability, ensuring high reliability even when dealing with subtle, nascent fault signatures that typically confound standard diagnostic architectures.

**Fig. 10:**
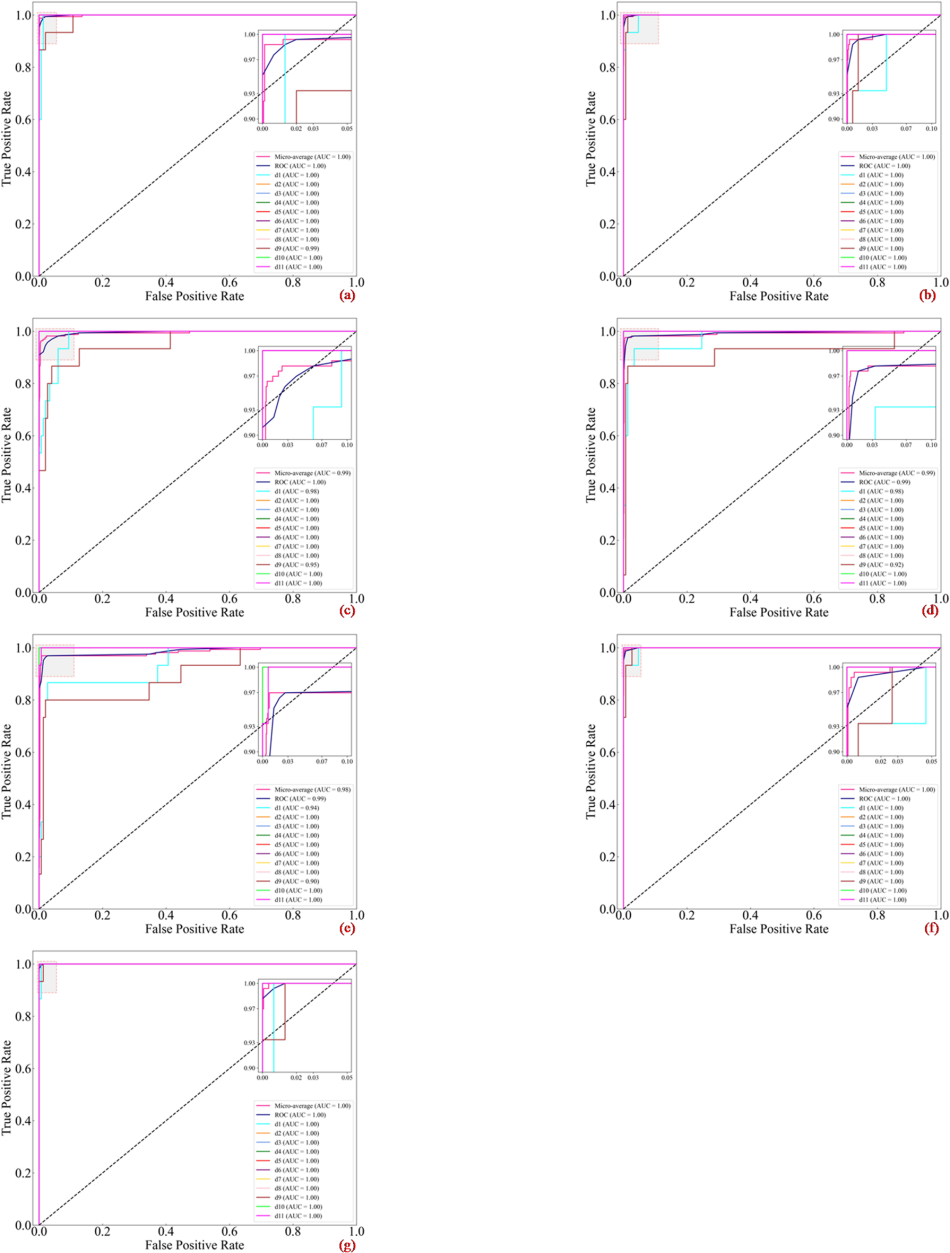
Precision-recall curves: (**a**–**g**) show the precision-recall curves for the seven methods on the self-built dataset. (**a**) CRIME-MDF-ACAM-ISimAM, (**b**) CRIME-TDF-ACAM-ISimAM, (**c**) CNN-LSTM, (**d**) WCNN-LSTM, (**e**) TSRF, (**f**) CNN-GRU-KAN, (**g**) Proposed method.

To evaluate the classification performance of the proposed model and the comparison model across varying decision thresholds, Receiver Operating Characteristic (ROC) curves were plotted for all diagnostic categories, as illustrated in Fig. 10. As shown in Fig. 10g, the curves for all eleven states lie in the extreme top-left corner of the coordinate space. This trajectory indicates that the CDAF maintains an exceptionally high True Positive Rate (TPR) while minimising the False Positive Rate (FPR) across all operating points. This exceptional balance of sensitivity and specificity stems from the model’s effective noise suppression, enabled by DPFD-JCSAM. By decoupling random noise from trend features, the framework prevents background fluctuations from triggering false alarms, thereby maintaining a low FPR even at sensitive decision thresholds. Quantitatively, the macro-average Area Under the Curve (AUC) for the proposed method reaches a near-perfect value of 1.0. While competing models such as CRIME-TDF-ACAM-ISimAM and CNN-GRU-KAN also achieve high macro-average AUC values, their individual class curves deviate more from the ideal top-left coordinate than those of the proposed integrated framework. This deviation implies that benchmark models lack the optimised decision boundaries provided by the CRIME algorithm. The proposed framework, through its cooperative evolutionary strategy, successfully maximises the classification margin between fault categories, ensuring higher confidence and stability in prediction. This comprehensive comparison of ROC trajectories and AUC values confirms the superior discriminative power and stability of the model under fluctuating decision confidence levels. These findings are highly consistent with the previously discussed performance metrics, confusion matrices, and *t*-SNE manifold visualisations, collectively establishing the CDAF as a robust and high-precision diagnostic tool.

#### Model robustness evaluation

**Fig. 11:**
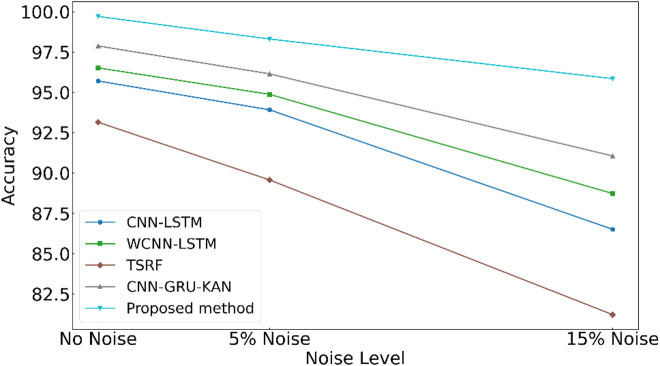
Accuracy curves for all methods under different noise levels.

To assess the diagnostic reliability and robustness of the framework under conditions approximating real-world industrial environments, the model was tested under varying levels of stochastic noise. Specifically, 5% and 15% Gaussian noise (representing moderate and severe environmental interference, respectively) were added to the thermal parameter test data. As summarised by the quantitative results in Table 5 and the accuracy degradation curves in Fig. 11, the proposed CDAF achieves an exceptional accuracy of 98.31% under mild noise. It retains a high diagnostic precision of 95.85% even under severe interference. In contrast, the second-best model (CNN-GRU-KAN) exhibits a clear performance inflexion point under high-amplitude noise; the accuracy gap between the proposed framework and the benchmark widens significantly from 2.20 to 4.97%. This divergence underscores that the proposed method’s advantages become increasingly prominent as environmental conditions deteriorate. In sharp contrast, the proposed framework overcomes these limitations through a unique dual-stage denoising and enhancement architecture: (1) the framework utilises an MLP-based dynamic filter, guided by the coupling of PCA-derived trend features and original mean values. This optimises the input space by precisely peeling off high-frequency noise while preserving the integrity of the underlying data manifold topology; (2) the ACAM-ISimAM module performs feature recalibration, dynamically locking onto and amplifying the essential weak fault signatures. This dual mechanism ensures effective noise-signal decoupling and refocusing of features. It maintains high intra-class compactness and distinct inter-class isolation even in the presence of extreme interference. These findings rigorously demonstrate the framework’s practical applicability to the diagnosis of subtle thermal faults in marine diesel engines, where high precision and unwavering robustness are essential in complex, real-world operating environments.

**Table 5:** Performance comparison of five methods under different noise levels.

Model	SNR = 5%				SNR = 15%			
	Accuracy (%)	Precision (%)	Recall (%)	F1-score (%)	Accuracy (%)	Precision (%)	Recall (%)	F1-score (%)
CNN-LSTM	93.92 ± 0.61	93.81 ± 0.68	94.05 ± 0.64	93.93 ± 0.56	86.51 ± 0.84	86.38 ± 0.91	86.73 ± 0.88	86.55 ± 0.73
WCNN-LSTM	94.88 ± 0.53	94.79 ± 0.59	95.02 ± 0.55	94.90 ± 0.48	88.73 ± 0.72	88.65 ± 0.78	88.89 ± 0.75	88.77 ± 0.64
TSRF	89.57 ± 0.92	89.81 ± 0.99	89.33 ± 0.95	89.57 ± 0.81	81.22 ± 1.15	81.53 ± 1.24	80.98 ± 1.21	81.25 ± 1.03
CNN-GRU-KAN	96.15 ± 0.42	96.22 ± 0.48	96.09 ± 0.45	96.15 ± 0.39	91.06 ± 0.58	91.15 ± 0.63	90.94 ± 0.61	91.04 ± 0.52
Proposed method	98.31 ± 0.35	98.31 ± 0.35	98.18 ± 0.33	98.24 ± 0.28	95.85 ± 0.45	95.92 ± 0.49	95.76 ± 0.48	95.84 ± 0.39

#### Performance comparison of optimisation algorithms

**Fig. 12:**
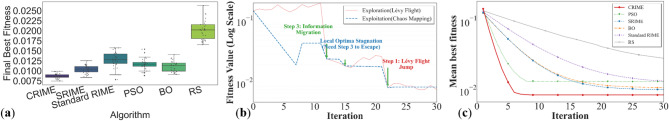
Optimisation performance and convergence of CRIME with various algorithms. (**a**) Box plots of different optimisation algorithms, (**b**) Search optimisation process of CRIME, (**c**) Convergence curves for varying optimisation algorithms.

To verify the superiority of the CRIME algorithm for automatic hyperparameter configuration, its performance was benchmarked against five representative optimisation strategies: SRIME, Standard RIME, Particle Swarm Optimisation (PSO), Bayesian Optimisation (BO), and Random Search (RS). The statistical results in Table 6 and the distribution patterns in Fig. 12a confirm the absolute advantage of the proposed algorithm in terms of both precision and stability. The convergence trajectories in Fig. 12c reveal that CRIME exhibits a unique, near-vertical initial descent within the first five iterations. The physical mechanism behind this rapid convergence is illustrated in Fig. 12b: the Lévy flight strategy provides the population with wide-area coverage, enabling it to jump across the search space and rapidly isolate high-quality candidate regions. While standard RIME and PSO encounter a performance bottleneck around the 10th to 15th generations, where the average error stagnates at 0.0128, CRIME effectively overcomes this local optima entrapment. At this critical juncture, the Information Migration mechanism, illustrated in Fig. 12b, facilitates co-evolution, forcing the population to break the convergence deadlock and escape extreme-value traps. This drives the CRIME curve in Fig. 12c to continue its descent throughout the mid-to-late iterations, finally stabilising at a global optimum of 0.0088. This synergistic cooperative mechanism, characterised by initial explosive exploration and mid-term dynamic breakout, results in the most compact distribution pattern in the box plots, shown in Fig. 12a, and the lowest standard deviation of 0.0007, as reported in Table 6. These findings strongly confirm the exceptional robustness and efficiency of the CRIME algorithm in configuring complex diagnostic models for marine diesel engines.

**Table 6:** Performance comparison of different optimisation algorithms across 30 independent runs.

Algorithm	Best	Mean	Worst	Std Dev
CRIME	0.0075	0.0088	0.0102	0.0007
SRIME	0.0081	0.0105	0.0131	0.0011
Standard RIME	0.0095	0.0128	0.0165	0.0019
PSO	0.0092	0.0117	0.0148	0.0015
BO	0.0085	0.0112	0.0135	0.0013
RS	0.0152	0.0195	0.0135	0.0025

#### Comparison of different explainability methods

To intuitively assess the model’s perceptual focus and diagnostic basis, we compared the proposed Fusion-CAM with two benchmark methods, Score-CAM and Grad-CAM++, across different network depths. This layer-by-layer visualisation shows how the model transitions from low-level feature identification to high-level semantic reasoning.

**Fig. 13:**
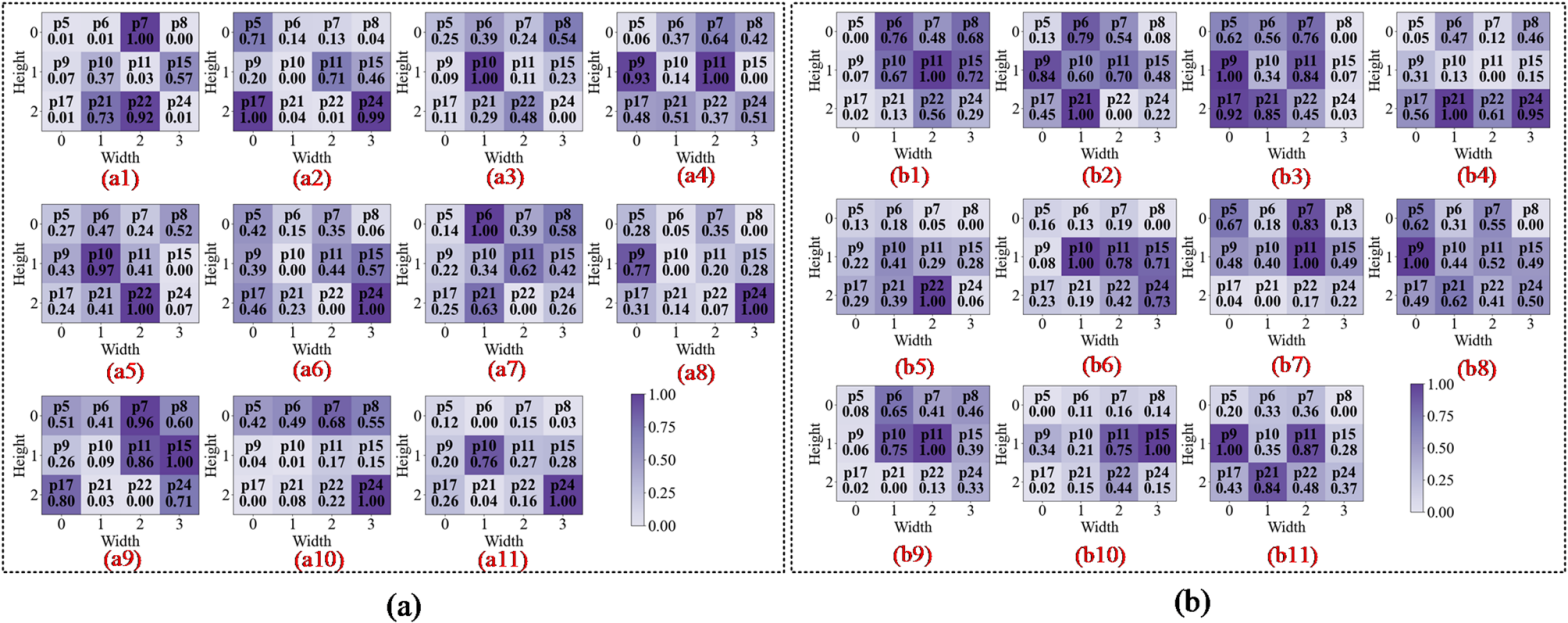
Grad-CAM + + feature response visualisation. (**a**) First convolutional fusion layer; (**b**) Final convolutional fusion layer.

In the first convolutional layer, the network primarily identifies low-level local features such as edges, gradients, and underlying textures. As illustrated in Figs. 13a8 and 14c8, the activation heatmaps for Score-CAM and Grad-CAM + + show coarse, undifferentiated regions that lack clear spatial focus. This suggests that these benchmark methods struggle to distinguish diagnostic saliency from background noise during the initial phase of feature extraction. In sharp contrast, Fusion-CAM demonstrates superior selective attention even at this shallow stage. As shown in Fig. 15e8, Fusion-CAM effectively suppresses irrelevant low-level features while significantly amplifying activation values for key parameters such as p24, with secondary focus on p9, p17, and p21. This suggests that the model, guided by Fusion-CAM, exhibits stronger discriminative focus in early layers, effectively suppressing noise-related redundancy and ensuring that a higher-quality feature stream is fed into the deeper architecture.

As the signal propagates to the second convolutional layer, the model integrates shallow features into a high-level semantic understanding of complex parameter combinations. In Figs. 13b8, 14d8, and 15f8 show that the resolution of the activation areas for Fusion-CAM is significantly higher than that of the benchmarks. While other methods yield scattered or blurred activation patterns, Fusion-CAM generates highly concentrated hotspots focused on parameters p7, p8, p11, and p22. The dynamic shift in parameter focus between layers provides a transparent diagnostic chain. For example, in the case of intake valve leakage (d8), the model initially prioritises parameters p9, p17, p21, and p24 in the shallow layer. However, in the deeper layer shown in Fig. 15f8, the focus shifts primarily to p11, with p7, p8, p15, and p22 serving as secondary contributors. This hierarchical transition reveals that while the shallow layer identifies raw anomalies, the deep network synthesises these into a definitive decision based on the high-level semantic role of p11. Similarly, for the non-uniform single-cylinder fuel supply fault (d5), shown in Fig. 15e5,f5, the model demonstrates that p24 is the primary driver, supported by a combination of p7, p8, p9, and p22. This layer-to-layer complementarity confirms that the CDAF does not rely on isolated data points but rather on a structured, physically traceable logic.

The comparative analysis confirms that Fusion-CAM is superior in terms of localisation precision, feature integrity, and explanatory fidelity. By providing strong visual evidence that aligns with thermodynamic principles, Fusion-CAM transforms the black box of deep learning into a transparent and credible diagnostic tool for marine engineering applications.

**Fig. 14:**
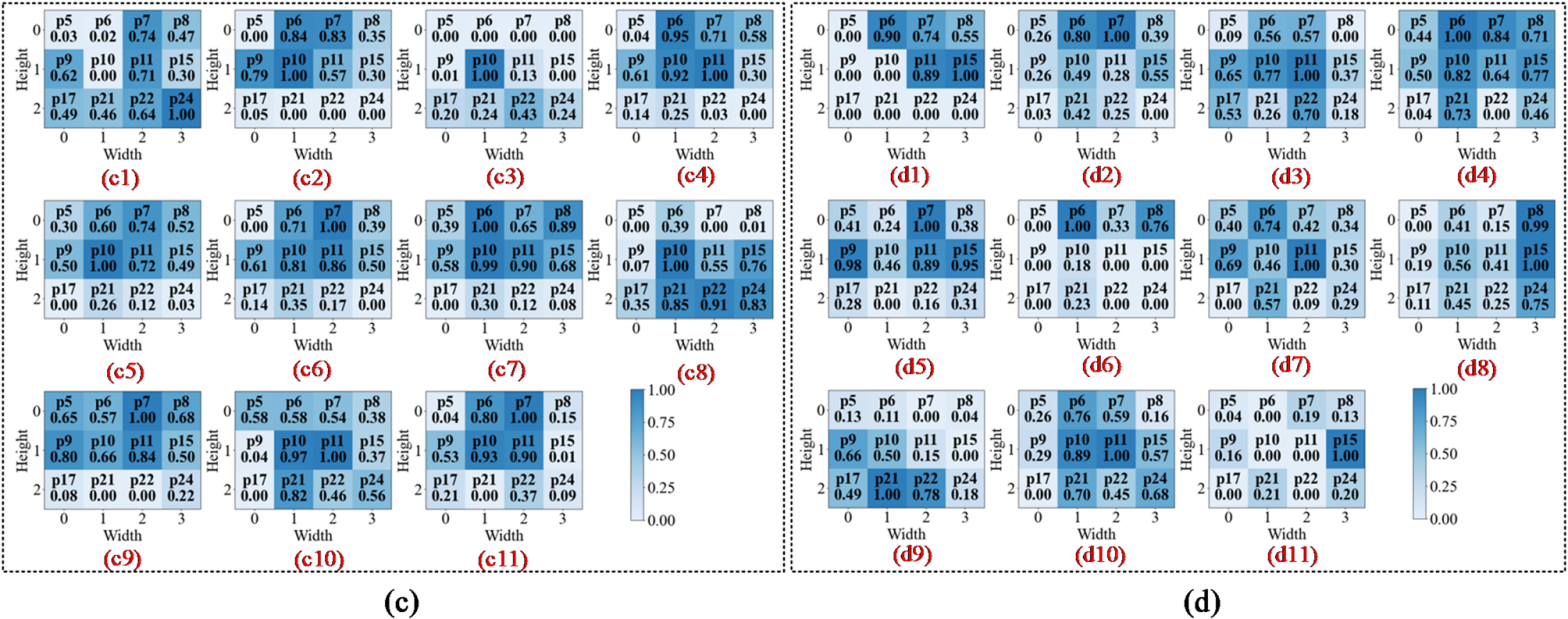
Score-CAM feature response visualisation. (**c**) First convolutional fusion layer; (**d**) Final convolutional fusion layer.

**Fig. 15:**
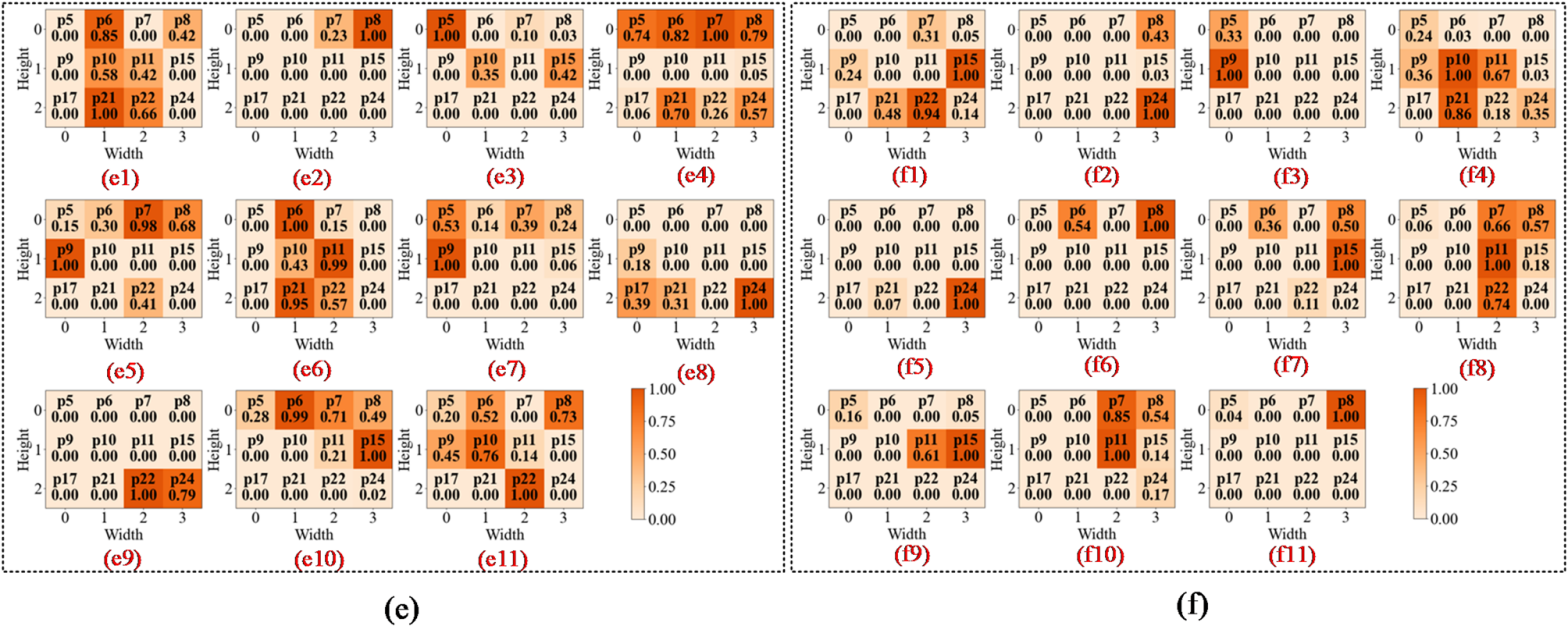
Visualisation of Fusion-CAM feature responses. (**e**) First convolutional fusion layer; (**f**) Final convolutional fusion layer.

To objectively and quantitatively evaluate the precision of various visualisation methods in identifying critical diagnostic features, a feature occlusion experiment was conducted. While visual analysis is valuable, it can be subjective; conversely, occlusion experiments provide a rigorous metric for verifying whether an explanation method has accurately identified the regions fundamental to the model’s decision-making process. The experimental procedure is as follows:

1. Heatmap generation: Feature activation heatmaps are generated for the test set using Score-CAM, Grad-CAM++, and the proposed Fusion-CAM.
2. Saliency selection: The top k = 20% regions with the highest activation intensity are designated as the key feature regions.
3. Feature perturbation: These identified regions are occluded in the original input data by padding them with zero values, effectively removing the information.
4. Performance re-evaluation: The occluded samples are reprocessed by the trained model, and the degradation in accuracy, precision, recall, and F1-score is recorded.

**Fig. 16:**
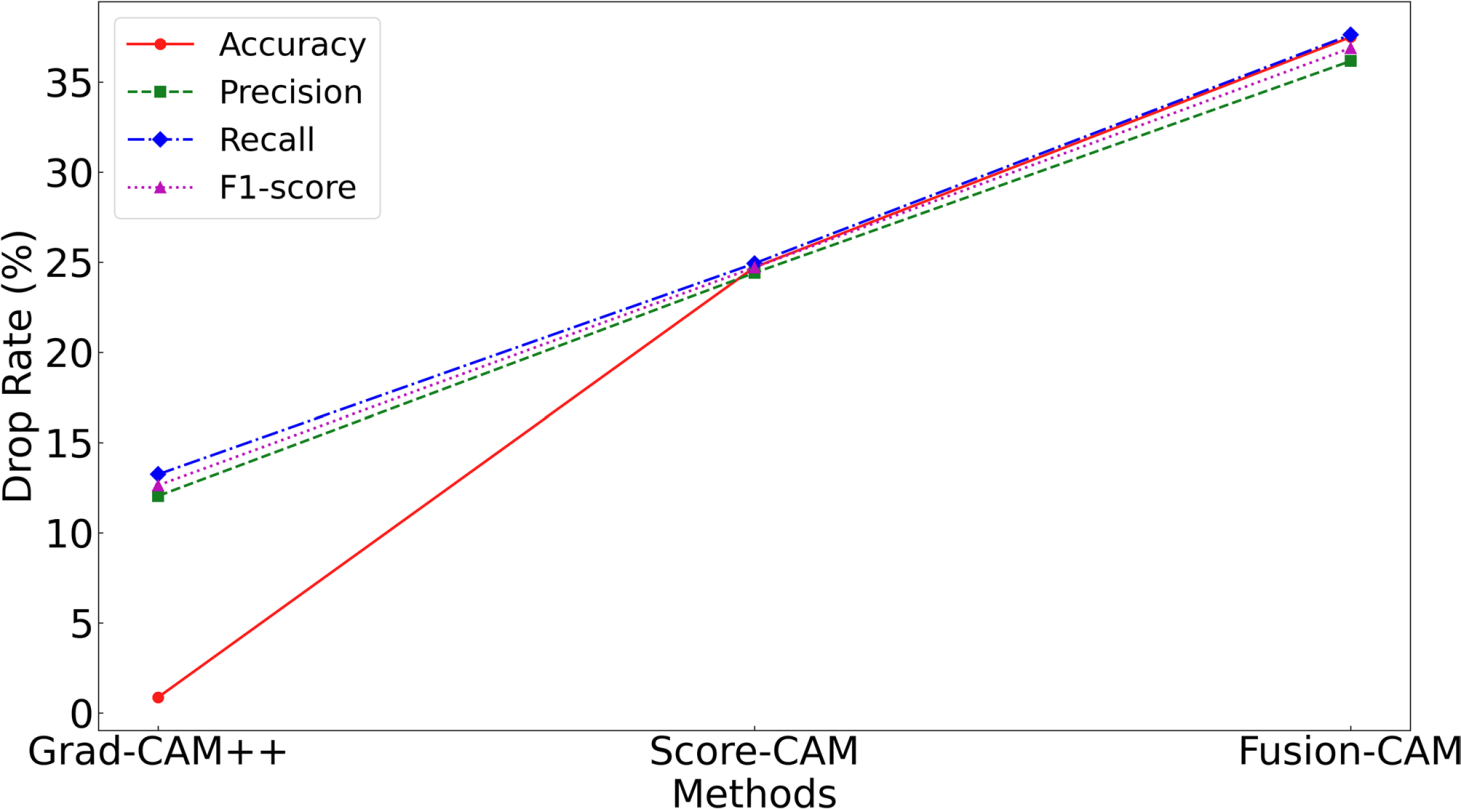
Performance degradation rate curves of occlusion sensitivity under different explainability methods.

The results of these experiments, summarised in Table 7, reveal a stark contrast in the localisation accuracy of the three methods. Before occlusion, the model serves as a stable baseline, with a diagnostic accuracy of 99.36%. Following occlusion of the regions identified by Grad-CAM++, accuracy decreases only slightly to 98.5%, suggesting that these regions are relevant but not critical to the final decision. Score-CAM shows a more pronounced effect, with accuracy dropping to 74.8%. The most dramatic performance collapse occurs after occlusion of the regions identified by Fusion-CAM. As illustrated in Fig. 16, diagnostic accuracy plummets to 62.1%, and the F1-score drops to 62.7%. This magnitude of performance decay, significantly larger than that of the benchmark methods, provides empirical evidence that Fusion-CAM successfully locks onto the core features that drive the model’s diagnostic logic. These results demonstrate that Fusion-CAM offers superior fidelity and is a highly reliable tool for identifying the physical roots of faults in complex marine diesel engines.

**Table 7:** Impact of key feature occlusion on diagnostic accuracy.

Explainable methods/metrics	Accuracy (%)		Precision (%)		Recall (%)		F1-score (%)	
	Before occlusion	After occlusion	Before occlusion	After occlusion	Before occlusion	After occlusion	Before occlusion	After occlusion
Grad-CAM++	99.36 ± 0.42	98.5 ± 0.22	99.48 ± 0.38	87.5 ± 0.63	99.24 ± 0.21	86.1 ± 0.77	99.35 ± 0.18	86.8 ± 0.58
Score-CAM		74.8 ± 0.71		75.2 ± 0.52		74.5 ± 0.83		74.8 ± 0.65
Fusion-CAM		62.1 ± 0.26		63.5 ± 0.44		61.9 ± 0.71		62.7 ± 0.53

**Fig. 17:**
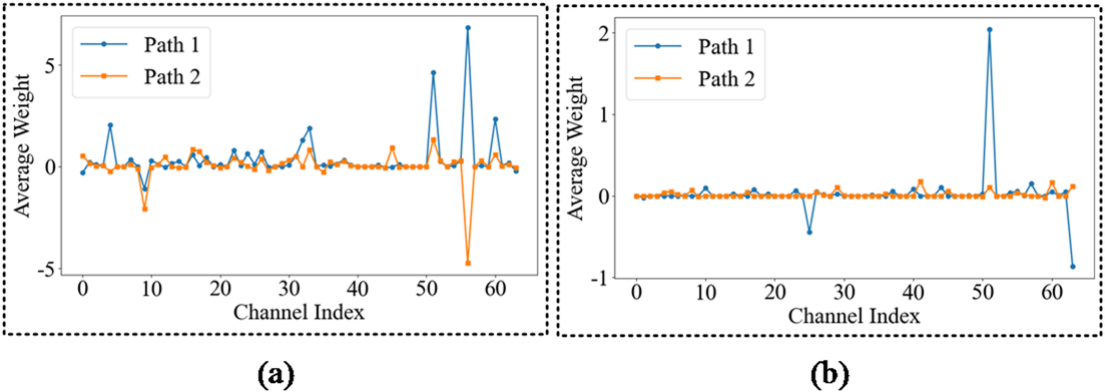
Channel weights for the trend-mean dual-pathway. (**a**) First feature fusion layer; (**b**) Last feature fusion layer.

Existing visualisation techniques, such as Score-CAM and Grad-CAM++, are primarily confined to spatial feature mapping within convolutional layers. They lack the capacity to delineate the specific gradient-based weight assignments for individual feature paths within a fusion architecture, typically relying on simplistic equal-proportion weighting. The proposed Fusion-CAM innovatively overcomes this limitation by quantifying the distinct channel weights of disparate paths within the fusion layer. Figure 17 illustrates the dual-path channel weight distributions for both the initial and final stages of the network, providing a clear visualisation of the model’s resource allocation between the two input streams. In the first convolutional layer, shown in Fig. 17a, the model exhibits a distributed weighting strategy. For the intake valve leakage (d8) state, channels 50 and 55 within Path 1 (Trend-based) and channels 9 and 55 within Path 2 (Mean-based) show elevated contributions. This suggests that at the shallow stage, the model relies on a balanced combination of dynamic evolutionary features and static magnitude indicators to identify the fault signature. In contrast, the deep convolutional layer, shown in Fig. 17b, reveals a more specialised attribution pattern. For the d8 state, while Path 1 shows significant contributions from channels 25, 51, and 62, the overall weight distribution indicates that Path 1 features (Trend-based) have evolved into the dominant factors influencing the final state prediction. Although the weight assigned to channel 2 in Path 1 is relatively small and fluctuates, the aggregate contribution of Path 1 outweighs that of Path 2 in this deeper stage. This granular analysis confirms that the Fusion-CAM can effectively trace how the importance of specific physical features shifts as they move through the deep network. By revealing that the diagnostic decision is ultimately driven by the mean-based path for this particular fault, Fusion-CAM provides engineers with a verifiable physical basis for confidence in the model’s high-level semantic conclusions.

#### Comparative explainability analysis of different network modules

**Fig. 18:**
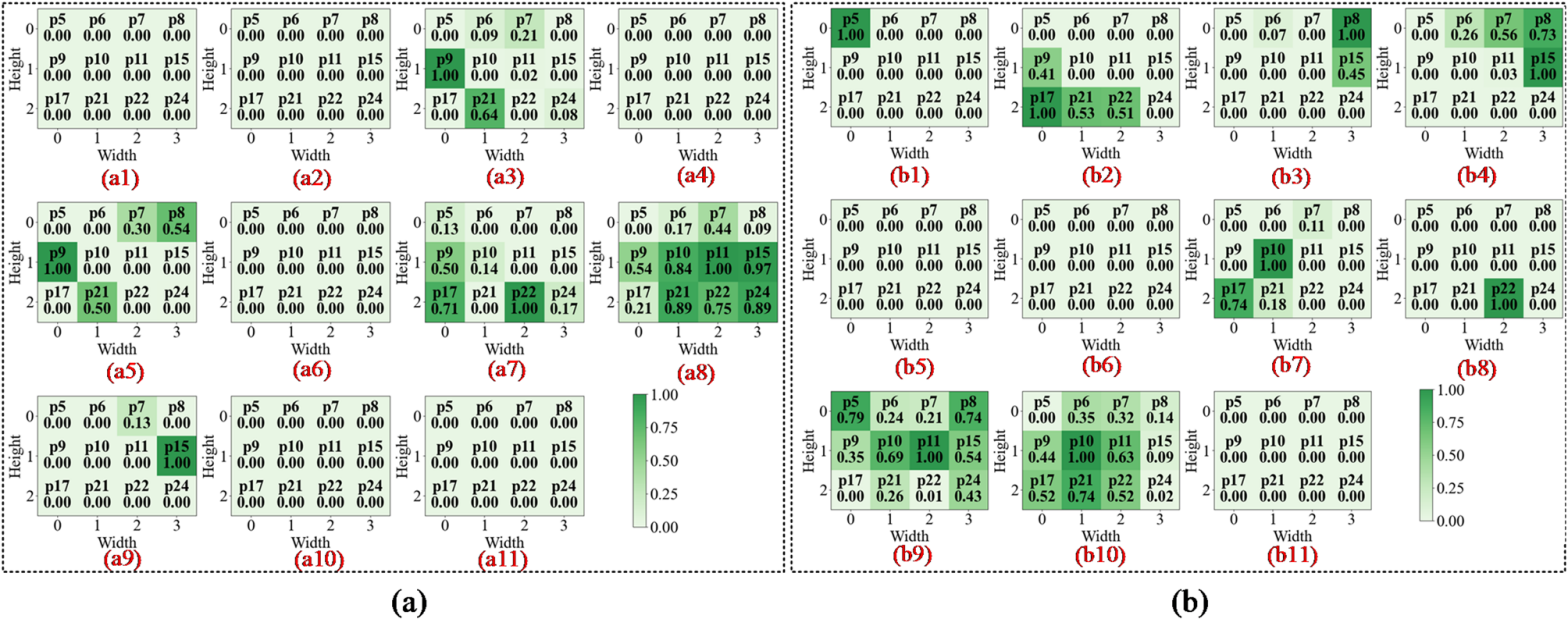
Visualisation of the Fusion-CAM feature response for the standard CNN module. (**a**) First convolutional fusion layer; (**b**) Last convolutional fusion layer.

To objectively verify the architectural superiority of the DPFD module over standard convolutional layers, we utilised Fusion-CAM to perform a comparative visualisation of feature activations across various health states. The results, illustrated in Fig. 15, reveal the fundamental difference in how these modules perceive complex thermal parameters. A critical finding emerges from the visualisation of the first and second convolutional fusion layers: the standard CNN module exhibits total deactivation when processing specific samples, such as those shown in Fig. 18a1,b5. In these instances, the activation heatmaps fail to show any discernible focus. This indicates that the static weights of a conventional CNN are inherently constrained to a fixed set of patterns learned during training; when confronted with subtle or non-standard fault manifestations, the layer fails to extract meaningful features, creating a bottleneck or short board that diminishes overall diagnostic accuracy. In sharp contrast, the DPFD module consistently extracts effective features across all tested states, including the challenging samples where the standard CNN failed. As shown by the clear, highly focused heatmaps in Fig. 15, DPFD generates strong activation signals even for nascent faults. The physical root of this superiority lies in its dynamic weight generation mechanism. Unlike static filters, the DPFD adaptively adjusts its parameters in real time, tailoring the filter response to the unique data characteristics of each input sample. This experiment provides more than just visual evidence; it fundamentally explains the model’s superior performance. By replacing rigid, static weights with a dynamic, data-driven filtering process, the CDAF ensures robust generalisation. It prevents the loss of critical diagnostic information, even under highly variable or noisy operating conditions.

**Fig. 19:**
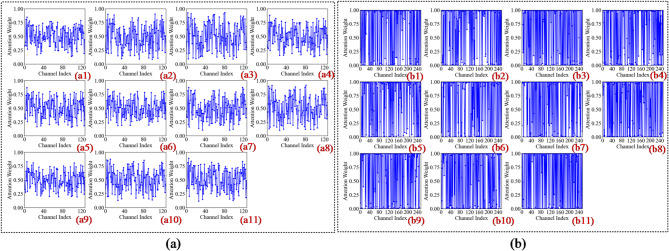
ACAM weight distribution curves for the conventional CNN module. (**a**) First feature fusion layer; (**b**) Last feature fusion layer.

**Fig. 20:**
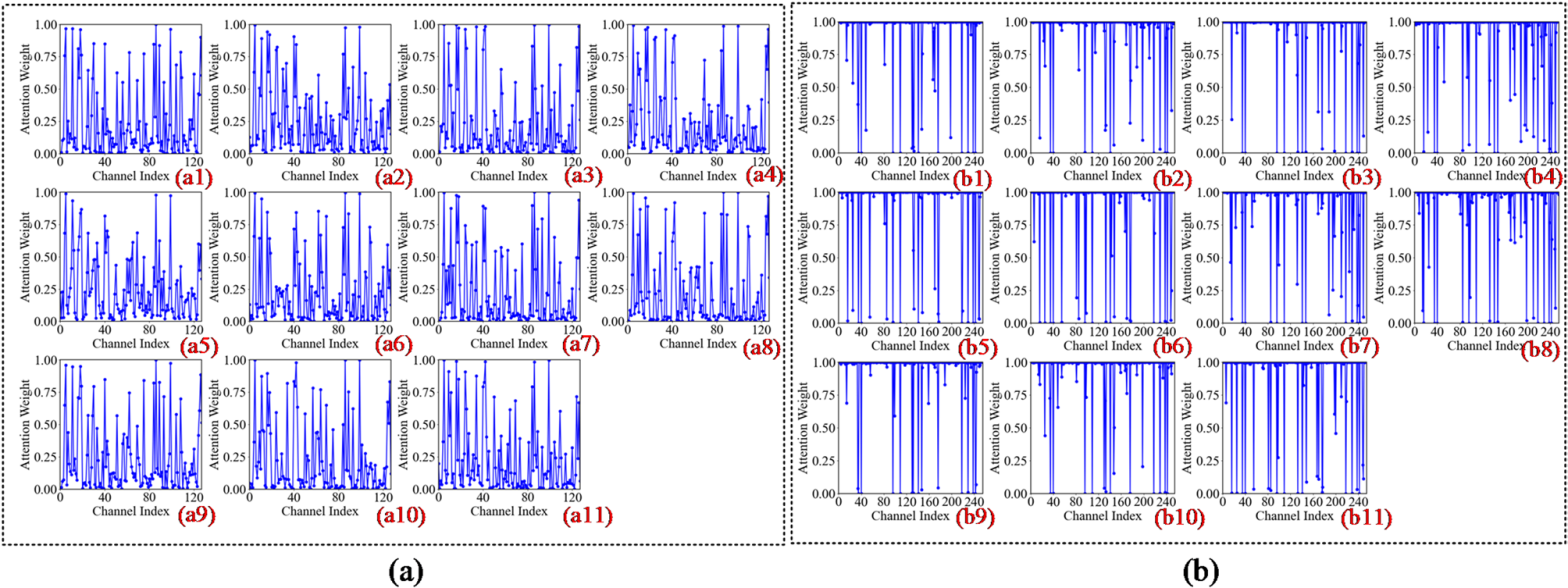
ACAM weight distribution curves for the DPFD module. (**a**) First feature fusion layer; (**b**) Last feature fusion layer.

To provide a more granular quantitative comparison between the standard CNN and the proposed DPFD module, we analysed the distribution of the channel attention weights applied to their respective output feature maps. This analysis quantifies the information entropy and the discriminative quality of the features generated by each architecture. The attention weight curves for the standard CNN module show a marked lack of discrimination. As illustrated in Fig. 19, the majority of weight values are densely clustered in the mid-range of 0.3 to 0.7, with a noticeable absence of extreme values approaching 0 or 1. This flat distribution indicates that, because the standard CNN utilises static kernels, the resulting feature maps are characterised by high information redundancy and inconsistent quality. The attention mechanism, therefore, cannot decisively prioritise specific channels, as the features themselves are not sufficiently distinct. In stark contrast, the DPFD module generates high-fidelity features that facilitate an efficient and decisive allocation of attention. As shown in Fig. 20, the weight curve for DPFD exhibits an evident polarisation phenomenon:

- Irrelevant suppression: The weights of a large number of non-contributory channels are effectively set to 0.
- Critical amplification: A select few highly relevant channels are elevated to a weight of nearly 1.0.

This high degree of weight sparsity demonstrates that the DPFD adaptively generates features with low information entropy. By tailoring filter responses to the specific characteristics of the input in real time, the DPFD ensures that the resulting feature space is highly correlated with the target fault state. This quantitative evidence explains why the DPFD-equipped achieves superior diagnostic precision; it transforms the blurred feature space of a standard CNN into a sparse, high-contrast representation that the attention modules can easily exploit.

**Fig. 21:**
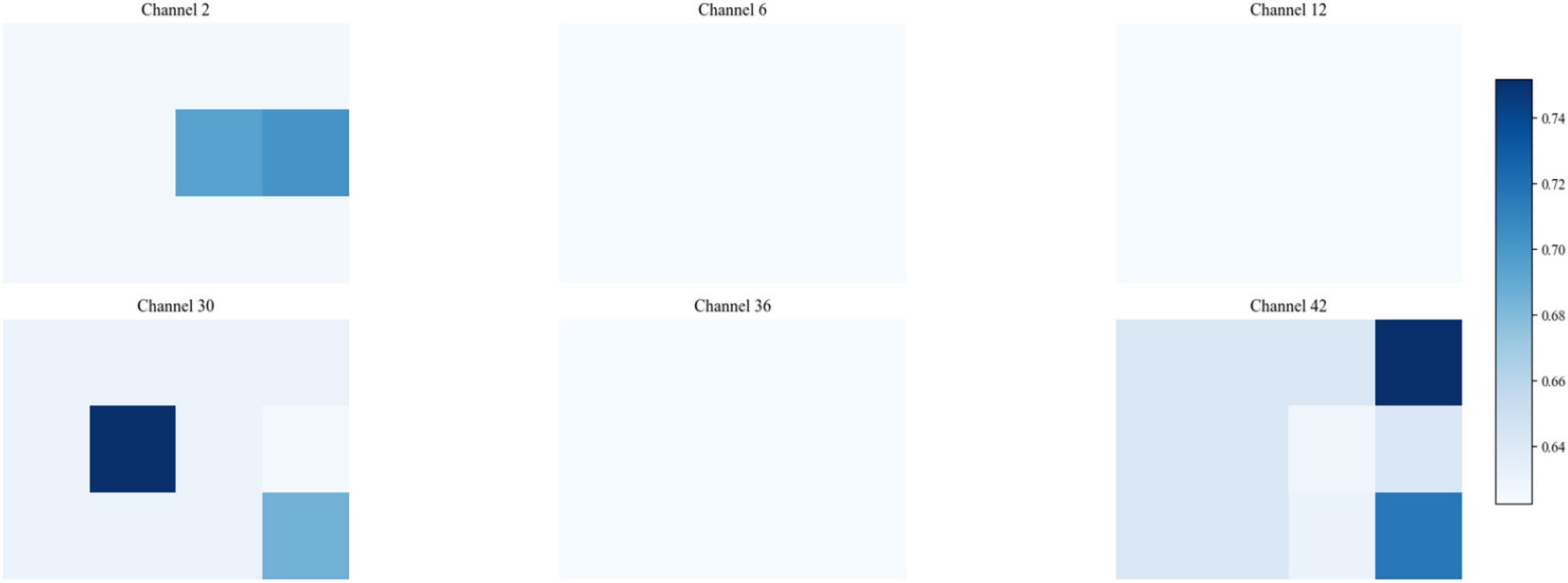
Visualisation of the standard CNN module under ISimAM.

**Fig. 22:**
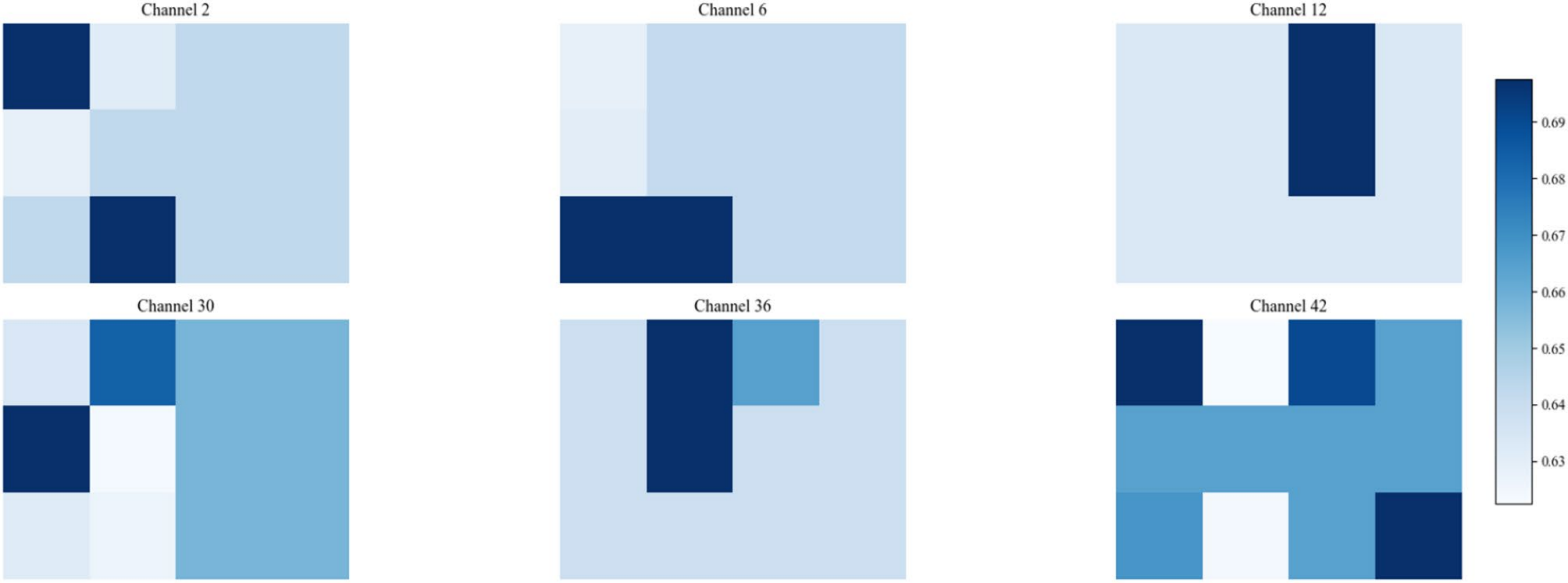
Visualisation of the DPFD module under ISimAM.

Beyond prioritising feature channels, the spatial attention mechanism plays a pivotal role in localising key diagnostic regions within the parameter grid. This section evaluates the spatial identifiability of the generated features by comparing the spatial attention heatmaps derived from both the standard CNN and the DPFD modules. As illustrated in Fig. 21, the heatmaps generated by the standard CNN module are characterised by diffuse, poorly defined activation patterns. Specifically, in channels 6, 12, and 36, the spatial attention weights are scattered across multiple parameter positions with low, uniform intensity. This failure to achieve a concentrated focal point suggests that the features produced by static kernels lack the necessary spatial discrimination to distinguish critical fault indicators from the surrounding data background. In stark contrast, the results shown in Fig. 22 demonstrate that the DPFD module significantly improves the spatial quality of the feature representations. The downstream spatial attention heatmaps exhibit highly focused, sparse activation patterns, with attention weights localised on a select few critical parameters. These regions form high-intensity hotspots (rendered in high contrast), providing an unambiguous signal for fault identification. This transformation from diffuse to sparse activation offers empirical proof that the dynamic filtering mechanism of the DPFD generates spatially distinguishable features. By suppressing spatial redundancy and sharpening the focus on anomalous parameter clusters, the DPFD ensures that the spatial attention module can operate with high precision, ultimately leading to more reliable and explainable diagnostic outcomes.

**Fig. 23:**
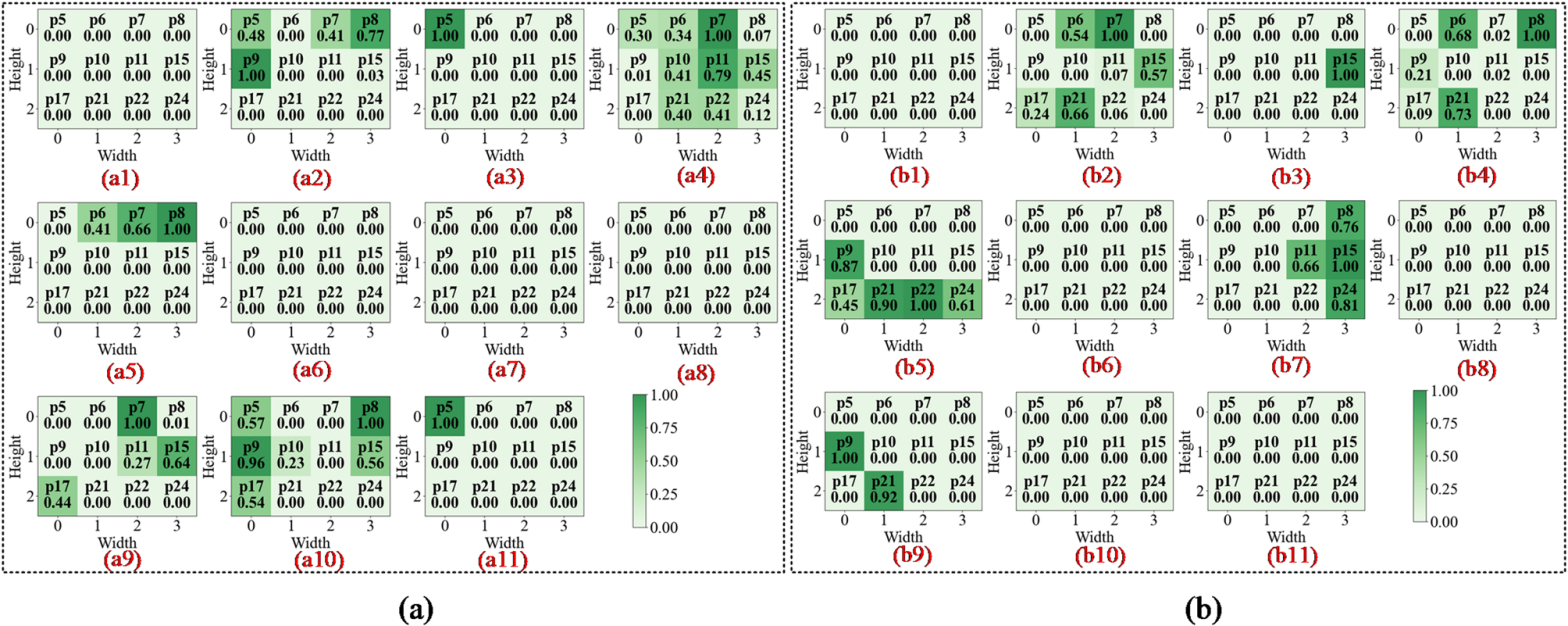
Visualisation of the CNN-LSTM model’s Fusion-CAM feature responses. (**a**) First convolutional fusion layer; (**b**) Second convolutional fusion layer.

**Fig. 24:**
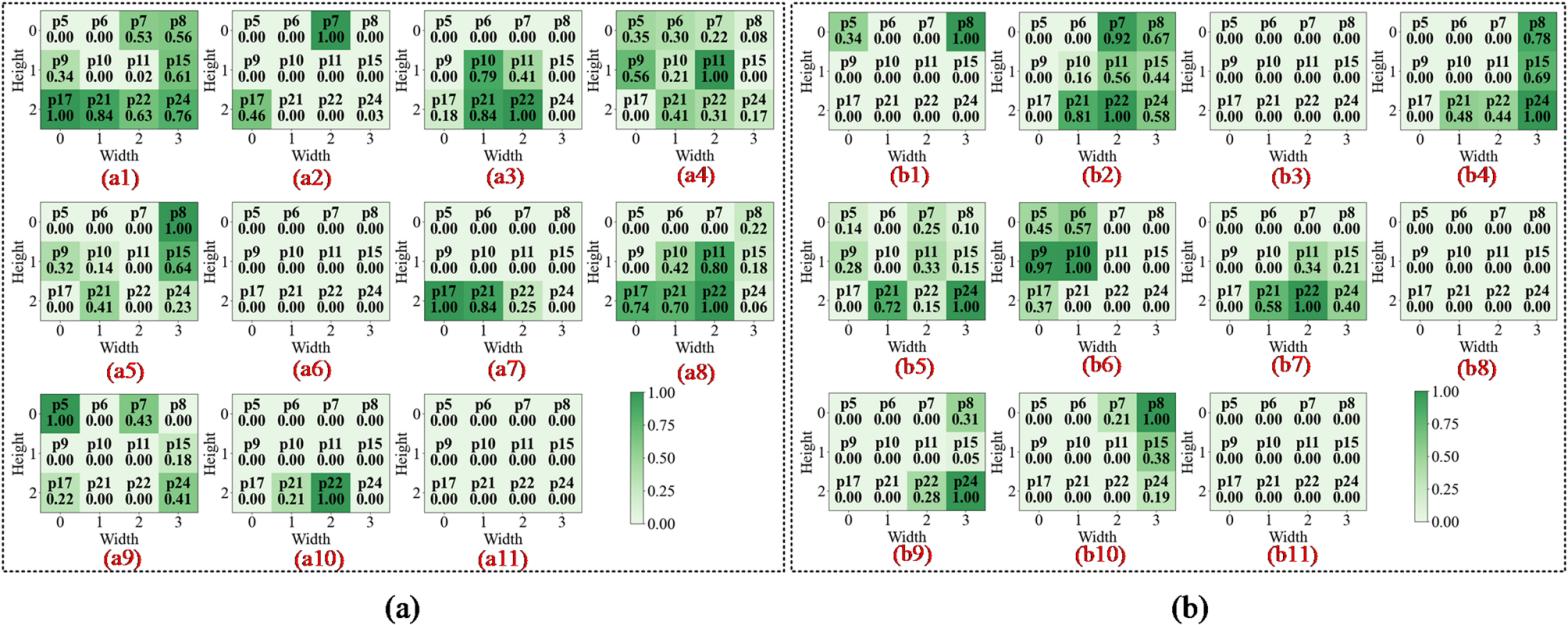
Visualisation of the WCNN-LSTM model’s Fusion-CAM feature responses. (**a**) First convolutional fusion layer; (**b**) Second convolutional fusion layer.

**Fig. 25:**
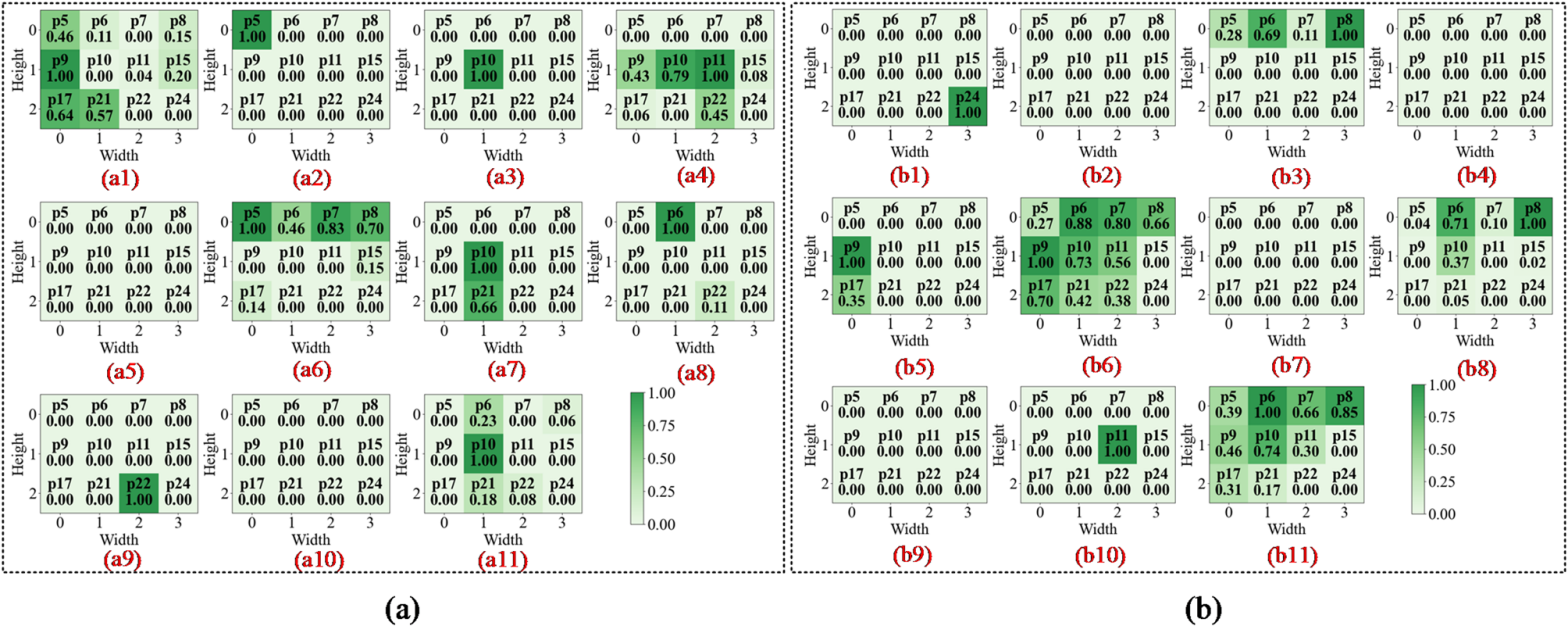
Visualisation of the CNN-GRU-KAN model’s Fusion-CAM feature responses. (**a**) First convolutional fusion layer; (**b**) Second convolutional fusion layer.

To intuitively assess the impact of model design on feature extraction efficacy, we conducted a comparative visual analysis of the latent feature maps generated by the proposed method and three representative models: CNN-LSTM, WCNN-LSTM, and CNN-GRU-KAN. Visual inspection of the benchmark methods reveals a common deficiency in feature representation. In both the shallow and deep convolutional layers, activation values are generally low and uniformly distributed, often resulting in the complete deactivation of features. As observed in Figs. 23 (states a1, b1), 24 (states a6, b3), and 25 (states a5, b2), many feature maps exhibit zero activation. This indicates an weak feature abstraction capability, with the models struggling to map specific input patterns to unique, discriminative descriptors. Furthermore, when subjected to weak fault signals embedded in noisy environments, these activation patterns are easily overwhelmed by stochastic interference, resulting in irregular activations and unstable representations. In sharp contrast, the feature maps extracted by the DPFD module exhibit a distinct, highly sparse and focused activation pattern. Within this dynamic framework, only a select subset of feature maps generates high-intensity activation values tailored to the specific characteristics of the input. In contrast, the remaining maps maintain a low-baseline state. This high-contrast representation demonstrates that the DPFD effectively extracts subtle fault signatures from background noise. These experimental findings provide robust evidence of the DPFD’s superior feature extraction capabilities. By ensuring high activation saliency even in the presence of subtle fault drifts, the DPFD architecture provides a stable and discriminative foundation that significantly outperforms existing hybrid and KAN-based architectures in marine diesel engine fault diagnosis (Figs. 24, 25).

## Conclusion

In this study, a novel Co-evolutionary Decoupled Attention Framework (CDAF) is proposed for the explainable weak thermal fault diagnosis of marine diesel engines. To overcome the local minima inherent in manual tuning, the framework is dynamically driven by the CRIME algorithm, which seamlessly integrates the DPFD module and JCSAM to isolate slow-varying thermodynamic degradation trends from intense stochastic sea-state noise and resolve fuzzy health-state boundaries. Demonstrating exceptional stochastic stability, CRIME achieved a superior global optimum of 0.0088 with a minimal standard deviation of 0.0007 and reached full convergence within the first 15 epochs. Consequently, the CDAF achieves an exceptional overall diagnostic accuracy of 99.71%. Notably, under severe 15% background noise interference, it maintains a robust accuracy of 94.85%, widening the performance gap over the second-best model (CNN-GRU-KAN) to 3.79% points.

Furthermore, the proposed Fusion-CAM marks a breakthrough in diagnostic transparency. Quantitative occlusion experiments unequivocally validate its precision: occluding core regions identified by Fusion-CAM triggered a dramatic accuracy collapse to 62.1%, a significantly steeper degradation than observed with Grad-CAM++ (98.5%) and Score-CAM (74.8%). For critical faults such as intake valve leakage, the framework successfully quantified specific channel contributions and traced the hierarchical synthesis of shallow features directly to primary diagnostic drivers (e.g., mean effective brake pressure). This fundamentally shatters the deep learning “black box” nature of the multi-source feature fusion layer, providing marine engineers with a highly transparent, evidence-based physical picture for reliable predictive maintenance.

While the framework exhibits exceptional performance on hybrid datasets, this study primarily focused on verifying the mechanism under class-balanced conditions. In real-world maritime operations, massive non-stationary datasets often exhibit significant sparsity and class imbalance. Future work will focus on developing unbalanced learning strategies tailored to complex operating conditions, ensuring that rare and weak thermal fault samples can be accurately identified in operational marine environments.

## Data Availability

All original code and part of the real-world marine diesel engine dataset have been deposited on GitHub and is publicly available at https://github.com/xiezaimi-png/Marine-diesel-engine-explainable-weak-thermal-fault-diagnosis.git.
